# Effects of the *in-utero* dicyclohexyl phthalate and di-*n*-hexyl phthalate administration on the oxidative stress-induced histopathological changes in the rat liver tissue correlated with serum biochemistry and hematological parameters

**DOI:** 10.3389/fendo.2023.1128202

**Published:** 2023-05-19

**Authors:** Duygu Aydemir, Mufide Aydogan-Ahbab, Nurhayat Barlas, Nuriye Nuray Ulusu

**Affiliations:** ^1^ School of Medicine, Department of Medical Biochemistry, Koc University, Istanbul, Türkiye; ^2^ Koç University Research Center for Translational Medicine (KUTTAM), Istanbul, Türkiye; ^3^ University of Health Sciences Turkey, Hamidiye Vocational School of Health Services, Istanbul, Türkiye; ^4^ Science Faculty, Department of Biology, Hacettepe University, Ankara, Türkiye

**Keywords:** DHP, DCHP, phthalates, oxidative stress, liver, biochemistry

## Abstract

Phthalates are widely used as plasticizers in the industry and are found in cosmetics, food and drink packaging, drugs, toys, households, medical devices, pesticides, personal care products, and paints. Phthalates exert endocrine disrupting and peroxisome proliferator effects in humans and wildlife associated with the pathogenesis of various diseases, including diabetes, obesity, infertility, cardiovascular diseases, metabolic syndrome, and cancer. Since phthalates are metabolized in the liver, which regulates the body’s energy metabolism, long or short-term exposure to the phthalates is associated with impaired glucose, lipid, and oxidative stress metabolisms contributing to liver toxicity. However, the impact of in-utero exposure to DHP and DCHP on liver metabolism has not been studied previously. Thus, in this study, we evaluated serum biochemistry parameters, hematological markers, histopathological changes, and oxidative and pentose phosphate pathway (PPP) metabolisms in the liver following in-utero DHP and DCHP administration, respectively, in male and female rats. We found increased relative and absolute liver weights and impaired triglyceride, alanine transaminase (ALT), lactate dehydrogenase (LDH), and alkaline phosphatase (ALP) levels upon dicyclohexyl phthalate (DCHP) and di-*n*-hexyl phthalate (DHP). Histopathological changes, including congestion, sinusoidal dilatation, inflammatory cell infiltration, cells with a pyknotic nucleus, lysis of hepatocytes, and degeneration of hepatic parenchyma have been observed in the liver samples of DHP and DCHP dose groups. Moreover, increased glutathione s-transferase (GST), glucose 6-phosphate dehydrogenase (G6PD), and glutathione reductase (GR) activities have been found in the liver samples of DHP and DCHP-treated rats associated with impaired pentose phosphate pathway (PPP) and oxidative stress metabolism. First time in the literature, we showed that in-utero exposure to DHP and DCHP causes liver damage associated with impaired oxidative stress metabolism in male and female rats. Our data may guide researchers and governments to regulate and restrict phthalates in industrial products.

## Introduction

Phthalates are used as plasticizers to increase the flexibility, durability, and processability of industrial products, including cosmetics, households, toys, medical devices, food packaging, drugs, pesticides, paints, and personal care products (1). Since people are exposed to phthalates daily, and these chemicals are widely detected in biological samples, phthalates are considered global pollutants, with 5.5 million tons of production worldwide in 2018, according to the OECD (Organization for Economic Co-operation and Development) (2). After exposure to phthalates *via* inhalation, digestion, or skin, they are metabolized by the liver, kidney, and intestines; however, some parts of these chemicals remain without metabolizing in the body and accumulate over time (3). Phthalates are detected in biological samples, including urine, blood, semen, fetus, and placenta in humans; thus, there is a growing concern about phthalate-induced adverse health effects in humans and wildlife since they exert endocrine disrupting effects by interfering with hormone metabolism (4).

Dicyclohexyl phthalate (DCHP) and di-*n*-hexyl phthalate (DHP) are plasticizers exerting endocrine-disrupting effects, including developmental and reproductive toxicity. DCHP was banned from cosmetics in China because of its adverse effects; however, below 0.1% DCHP in toys and personal care products is allowed in the USA (5). DHP and DCHP are listed as substances of severe concern and reported as toxic to reproduction in the EU officially; also, both chemicals are considered the most potent phthalates (6). DCHP and DHP are found in food packages, PVC, paper wrappers, floorings, canvas traps, drugs, and paper labels; they can be quickly released from industrial products since they are not covalently bound to polymers (7). Both phthalates are detected in the human placenta, semen, cord blood, serum, and blood and are associated with impaired reproductive and developmental function in humans and wildlife (8).

The placenta is constitutively exposed to phthalates during pregnancy, and these EDCs can cross the placental barrier leading to decreased low birth, preterm birth, miscarriages, and decreased fecundity (9). The placenta supports fetus growth by supplying oxygen, nutrients, hormones, and signaling molecules as an endocrine organ associated with fetal development (10). Placenta synthesizes steroid hormones, including estriol (E3), estradiol (E2), and progesterone, for maintenance of the pregnancy; however, phthalates decrease estrogen and progesterone levels in the placenta via activating PPAR-α leading to the altered fetal growth and development. Furthermore*, in-utero* exposure to phthalates has adverse health effects in adulthood, including diabetes, metabolic disorders, obesity, cancer, and cardiovascular diseases. On the other hand, there is no information about the biochemical and histopathologic effects of prenatal phthalate exposure in adulthood associated with liver metabolism (11).

Phthalates are metabolized in the liver *via* hydrolysis, oxidation, and conjugation processes depending on the type of the chemicals; then, they are excreted in urine and feces (12). The liver regulates lipid, oxidative stress, glucose, detoxification, energy, and protein metabolism. Phthalate exposure leads to hepatic cell apoptosis, liver fibrosis, insulin resistance, dysregulated cholesterol metabolism, inflammatory cell infiltration, altered hepatocyte morphology, enhanced oxidative stress, and lipid metabolisms contributing to the pathogenesis of diabetes, metabolic syndrome, cardiovascular diseases, obesity, cancer, and neurological disorders (13). Decreased antioxidant capacity, increased oxidative stress, impaired glucose, and energy metabolisms have been reported in the liver of orally phthalate-administered rats such as DEHP, DBP, MEHP, diisobutyl phthalate (DIBP), and diisononyl phthalate (DINP) (14–16). Since biochemical and histopathological effects of the *in-utero* DHP and DCHP exposure on adult rats have not been studied before, in this study, we have evaluated the impact of the *in-utero* exposure of DCP and DCHP on the liver metabolism in adult rats associated with oxidative stress metabolism-induced histopathological changes first time in the literature.

## Materials and methods

### Materials

DHP (CAS No. 84-75-3) with the purity of 97% and DCHP (CAS No. 84-61-7) with the purity of 99% was supplied by Alfa Aesar and Aldrich Chemistry (Ankara, Turkey), respectively. Oxidized glutathione (GSSG), nicotinamide adenine dinucleotide phosphate (NADP+), 6-phosphogluconate (6-PG), glucose-6-phosphate (G6P), reduced glutathione (GSH), reduced nicotinamide adenine dinucleotide phosphate (NADPH + H+), magnesium chloride (MgCl_2_), sodium phosphate monobasic and dibasic, tris base and cOmplete™ Protease Inhibitor Cocktail were obtained from Sigma-Aldrich (St. Louis, MO, USA).

### Animal laboratory

The female and male Wistar albino *Rattus norvegicus* rats were purchased from the Experimental Animals Production Center, Hacettepe University in Ankara, Turkey. Permission required for the studies was obtained from Hacettepe University Experimental Animals Ethics Committee with the number 2005/46-3. Before the animal experiments started, the rats brought to the laboratory were kept under suitable conditions for seven days to get used to the laboratory environment. Female and male rats were mated overnight, and the gestational day 0 (GD0) was determined when sperm was detected in the vaginal lavage. All rats were housed in polycarbonate cages in a room maintained in a 12-h light/dark cycle with a temperature of 21 ± 2°C and relative humidity of 50 ± 5% and given a standard rat diet and tap water *ad libitum*.

### Experimental design and treatments

Before mating the male and female, female rats were checked for the stage of the oestrous cycle. Females showing a late proestrus or oestrous smear are placed with a male in the cage for mating overnight. In the morning, vaginal plug existence or sperm in the vaginal lavage smear was used to confirm pregnancy, which was assumed as gestational day 0 (GD0). Pregnant rats (dams) were divided into a control group administered only with corn oil (n = 10) as the vehicle and treatment groups administered with DHP and DCHP prepared in the corn oil (n=60), respectively. DHP and DCHP administration was performed at dosages of 0 (corn oil=vehicle) (n=10), 20 (n=10), 100 (n=10), and 500 mg/kg (body weight)/day (n=10) prepared in corn oil by gavage from GD 6 to GD 19. The DHP and DCHP solutions were prepared daily and adjusted according to the weight of each rat, with a dosing volume of 0.25 ml in each group. DHP and DCHP dosages have been chosen without exceeding the acute oral median lethal dosages as 29.6 mg/kg/day for DHP and >40 g mg/kg/day for DCHP for rats (17). The low-dose level has been determined depending on the no observed adverse effect level (18), and the high-dose group has been calculated based on the lowest-observed-adverse-effect level of 500 mg/kg/day according to the literature (19).

The dams in the vehicle control group received corn oil in amounts equal to the experimental groups. After delivery, all pups had grown with dams for one month, then male and female pups were separated. Rats were housed in polycarbonate cages and fed a standard rat diet and tap water *ad libitum*. Male and female rats were grown and divided into three groups according to age prepubertal (postnatal day (PD) 20), pubertal (PD 32), and adult (PD 90). In our study, we have used adult rats as follows groups; control (n=10), 20 mg/kg/day DHP (n=10), 100 mg/kg/day DHP (n=10), 500 mg/kg/day DHP (n=8), 20 mg/kg/day DCHP (n=10), 100 mg/kg/day DCHP (n=10), 500 mg/kg/day DCHP (n=10) dose groups. At necropsy, each animal was weighed and sacrificed under ether anesthesia. Liver, kidneys, spleen, stomach, heart, thymus, brain, and lung tissues were collected. Each tissue was weighed to calculate each animal’s relative and absolute organ weights before freezing tissues in liquid nitrogen. The Approval of Ethics Committee of Hacettepe University, with the number B.30.2.HAC.0.01.00.05 approved all experimental procedures and animal use.

### Hematological analysis

Whole blood was directly taken from the heart of male and female rats with sterile syringes under anesthesia and placed into EDTA-containing blood tubes to measure hematological parameters. Leukocyte, lymphocyte, monocyte, granulocyte, erythrocyte, mean corpuscular volume (MCV), hematocrit, mean corpuscular hemoglobin (MCH), mean corpuscular hemoglobin concentration (MCHC), hemoglobin, ratio distribution width of red blood cells-standard deviation (RDW-SD), red cell distribution width - coefficient of variation (RDW-CV), erythrocyte, platelet, mean platelet volume (MPV), procalcitonin (PCT), platelet distribution width (PDW) and methylenetetrahydrofolate reductase (MTFHR) were measured with a hematology analyzer (MS9-5).

### Serum biochemical analysis

Whole blood was taken from the heart and placed into the heparin tubes. Samples were centrifuged at 3000 rpm for 15 min to isolate serum. Creatinine, urea, glucose, alanine aminotransferase (ALT), alkaline phosphatase (ALP), total protein, albumin, gamma-glutamyl transferase (GGT), aspartate aminotransferase (AST), lactate dehydrogenase (LDH), total cholesterol and triglyceride amounts were measured with a Shimadzu CL-770 clinical spectrophotometer by using Audit Diagnostic kit.

### H&E staining of liver samples

Liver samples were collected after sacrification and weighed. Liver samples were fixed in Bouin’s solution for 8 h. Fixed samples were embedded in paraffin, cut to 4-μm thickness, and stained with Harris hematoxylin and eosin (H&E). All slides were examined with an Olympus BX51 light microscope to evaluate histopathological changes in the liver. Images of slides were captured using Bs200prop software, histopathological changes of each slide were recorded, and statistical analysis was performed to compare treated and control groups.

### Tissue lysate preparation

30-50 mg of liver tissue sample was homogenized in the sodium phosphate buffer (Na_2_PO_4_) containing protease inhibitor cocktail tablet (freshly added) with an Ultra Turrax homogenizer with an S18N-10G probe (IKA, Königswinter, Germany). Liver homogenates were centrifuged in a Beckman Coulter ultracentrifuge (Fullerton, CA, USA) at 105,000×g at 4°C for 60 min, and the supernatant was collected to evaluate enzyme activities.

### Evaluation of the glucose 6-phosphate dehydrogenase activity

The reaction mixture was prepared with 0.2 mM NADP^+^, 0.6 mM glucose-6 phosphate (G6P), and 10 mM MgCl_2_ in 100 mM Tris-HCl buffer (pH 8.0) was used to evaluate G6PD activity in the liver samples. Each enzyme activity was measured by Synergy H1 BIOTEK spectrophotometer *via* following NADPH production at 340 nm at 37°C for 60 s.

### Measurement of the 6-phosphogluconate dehydrogenase activity

The same reaction mixture evaluated 6-PGD activity in the liver tissue with G6PD, except 0.6 mM 6-phosphate gluconate (6PG) is used as substrate instead of G6P. Assays were duplicated, and specific enzyme activity was given as the number of units/mg of protein.

### Evaluation of glutathione reductase activity

The liver lysate was incubated with 100 mM sodium phosphate buffer (pH 7.4), 1 mM GSSG, and 0.2 mM NADPH to measure GR activity. A unit of activity (U) is defined as the amount of enzyme that catalyzes the oxidation of 1 μmol of NADPH in 1 min under these conditions.

### Evaluation of glutathione s-transferase activity

Glutathione s-transferase enzyme activity in the liver lysates was evaluated by measuring the conjugation of GSH with 1-chloro-2,4-dinitrobenzene (CDNB). The reaction mixture was prepared with 0.2 M sodium phosphate buffer (pH 6.5), 25 μl of 20 mM CDNB, 25 μl of 20 mM GSH, and liver lysate to measure GST activity.

### Soluble protein determination in the liver samples

Soluble protein concentration in all samples was evaluated by a Pierce BCA Protein Assay kit (Thermo Scientific, USA) using albumin as standard according to the kit instructions. Samples were pipetted into the 96 well plates and read at 562 nm using Synergy H1, BIOTEK.

### Evaluation of total SOD levels *via* ELISA

Cytosolic lysates of liver samples were used to evaluate cytosolic total SOD levels using the Abbkine Rat SOD1 ELISA kit (Abbkine, China). Samples were pipetted in the 96-well plate of the ELISA kit, and after incubation with HRP-conjugated antibody, wells were washed and treated with chromogen solutions according to the kit instructions (KTE101023, Abbkine, China). After the experimental procedure, the samples’ absorbance was read at 450 nm.

### Evaluation of FASN levels *via* ELISA

Fatty acid synthase (FASN) levels in the liver samples were measured *via* Enzyme-Linked Immunosorbent Assay (ELISA). 100 µl of liver lysate samples were pipetted into the 96-well-precoated plates, and the assay was carried out according to the kit instructions (SEC470Ra, USCN, China). After the experimental procedure, the samples’ absorbance was read at 450 nm.

### Statistical analysis

Statistical analysis of body weights, organ weights, serum biochemistry parameters, hematological biomarkers, and enzyme activities was performed by GraphPad Prism software (9.0) using one-way or two-way analysis of variance (ANOVA) followed by a Tukey’s *post hoc* test for multiple comparisons. The results are represented as each set’s mean ± Standard deviation (SD), and p-value<0.05 was considered significant. On the other hand, the histopathological effects were evaluated *via* SPSS 18.0 for Windows by independent-sample *t*-test for equal or unequal variances as appropriate. Levene tests were used to assess data for normality and homogeneity, where Fisher’s exact test was used to compare histopathological changes. All values presented in the text are mean ± standard deviation (SD), and a p-value<0.05 was considered significant.

## Results

### Impact of the DHP and DCHP administration on the absolute and relative organ weights of male and female rats

DHP and DCHP were administered *in utero* to the pregnant rats, and after birth, male and female rats were divided into the prepubertal (PD 20), pubertal (PD 32), and adult (PD 90) groups. There was no significant change in maternal body weight in DHP and DCHP-administered groups compared to the control animals (20). Before sacrification, each animal was weighed, and body weights were recorded. The final body weight of adult female rats significantly increased in all DHP and DCHP administered groups compared to the control, respectively, except the 100 mg/kg/day DHP dose group (**Supplementary Table 1**). On the other hand, body weights did not show any significant differences between control and DHP or DCHP-treated groups in male rats (**Supplementary Table 2**). The liver, kidney, brain, thymus, spleen, stomach, heart, and lung tissues of male and female rats were collected and weighed following sacrification (**Supplementary Tables 1**, **2**), where relative organ weight was calculated as an organ (mg)/body weight (g) (**Table 1**).

**Table 1:** Relative organ weights (mg/g) of adult male and female rats were calculated following sacrification to evaluate the impact of *in utero* administration of DHP and DCHP at the dosages of 20, 100 or 500 mg/kg/day respectively.

n	Control			DHP (mg/kg/day)									DCHP (mg/kg/day)								
				20			100			500			20			100			500		
	10			10			10			10			8			10			10		
Relative Weight (mg/g)																					
Male																					
*Liver*	37	±	1.7	36	±	2	37.2	±	1.3	35.2	±	1.7	38	±	3	38.4	±	2 **^a^**	36.4	±	1.3
*Kidney*	3.6	±	0.3	3.2	±	0.1 **^b^**	3.5	±	0.2	3.5	±	0.2	3.5	±	0.2	3.5	±	0.1	3.4	±	0.3 **^c^**
*Spleen*	2	±	0.2	1.45	±	0.1 **^d,e,f,g,h,i^**	1.82	±	0.2	1.83	±	0.2	1.96	±	0.2	2	±	0.1	1.9	±	0.2
*Stomach*	5.2	±	0.4	5.5	±	0.6	5.1	±	0.5	4.8	±	0.4	4.9	±	0.4	4.8	±	0.4	5.1	±	0.5
*Heart*	2.7	±	0.1	2.9	±	0.2	3	±	0.2	2.6	±	0.1 **^j,k^**	2.8	±	0.3	3.0	±	0.3	2.8	±	0.1
*Thymus*	1.6	±	0.3	1.6	±	0.2	1.6	±	0.4	1.8	±	0.2	1.5	±	0.2	1.8	±	0.2	1.6	±	0.2
*Lung*	5.5	±	1	4.4	±	0.4 **^l,m^**	5.4	±	0.6	6.1	±	2.2	5.1	±	0.6	6.2	±	1	5.9	±	0.7
*Brain*	7.3	±	0.7	7.0	±	0.5 **^n^**	7.1	±	0.3	6.9	±	0.3	7.4	±	0.7	6.6	±	0.3	6.8	±	0.3
Female																					
*Liver*	33.5	±	2.4	39.7	±	3.2 **^o,p^**	36.4	±	4.1	36.2	±	3.5	35	±	2	36.9	±	2	37.4	±	2.4
*Kidney*	3.5	±	0.1	3.4	±	0.2	3.5	±	0.2	3.7	±	0.2 **^q,r,s,t^**	3.5	±	0.2	3.5	±	0.1	3.6	±	0.2
*Spleen*	2.3	±	0.3	1.87	±	0.5**^u^**	2.2	±	0.3	2.2	±	0.4	2.1	±	0.2	2.5	±	0.2	2.2	±	0.2
*Stomach*	5.8	±	0.5	6.4	±	0.4	6.3	±	0.4	5.1	±	0.2	5.9	±	0.3	4.9	±	1.9**^v,w^**	5.9	±	0.4
*Heart*	3	±	0.1	3.2 **^y^**	±	0.2	3	±	0.1	3.4	±	0.3	2.8	±	0.1**^x,z,A B^**	3.3	±	0.3	3.1	±	0.1
*Thymus*	2	±	0.1	2.1	±	0.5	2.2	±	0.3	2.3	±	0.5	2	±	0.2	2	±	0.3	2.5	±	0.5
*Lung*	6.4	±	1.1	4.5	±	1.8**^C,D,E^**	6.2	±	1.1	5.8	±	1	6.4	±	1.5	7	±	1	7.2	±	0.9
*Brain*	10.1	±	0.5	8.8	±	0.5 **^F^**	10	±	0.9**^I,J,K,L,M^**	9.6	±	0.8	9.4	±	0.6	8.3	±	0.4**^G,N,O^**	8.7	±	0.7**^H^**

a statistically different from 500 mg/kg/day DHP dose group (p=0.0229), statistically different from control group (p<0.0001), statistically different from control group (p=0.0194), statistically different from control group (p<0.0001), statistically different from 100 mg/kg/day DHP dose group (p=0.0102), statistically different from 500 mg/kg/day DHP dose group (p= 0.0149), statistically different from 20 mg/kg/day DCHP dose group (p=0.0003), statistically different from 100 mg/kg/day DCHP dose group (p<0.0001), statistically different from 500 mg/kg/day DCHP dose group (p=0.0012), statistically different from 100 mg/kg/day DHP dose group (p=0.0049), statistically different from 100 mg/kg/day DCHP dose group (p=0.0189), statistically different from 500 mg/kg/day DHP dose group (p=0.0280), statistically different from 100 mg/kg/day DCHP dose group (p= 0.0083), statistically different from 100 mg/kg/day DCHP dose group (p=0.0372), statistically different from control group (p= 0.0026), statistically different from 20 mg/kg/day DCHP dose group (p=0.0345), statistically different from 20 mg/kg/day DHP dose group (p=0.0209), statistically different from 100 mg/kg/day DHP dose group (p=0.0411), statistically different from 20 mg/kg/day DCHP dose group (p=0.0039), statistically different from 100 mg/kg/day DCHP dose group (p=0.0345), statistically different from 100 mg/kg/day DCHP dose group (p=0.0044), statistically different from 20 mg/kg/day DHP dose group (p=0.0457), statistically different from 100 mg/kg/day DHP dose group (p= 0.0450), statistically different from control group (p=0.0130), statistically different from 20 mg/kg/day DCHP dose group (p=0.0118), statistically different from 100 mg/kg/day DHP dose group (p=0.0470), ^A^ statistically different from 20 mg/kg/day DHP dose group (p<0.0001), ^B^ statistically different from 100 mg/kg/day DCHP dose group (p=0.0010), ^C^ statistically different from 20 mg/kg/day DCHP dose group (p< 0.0304), ^D^ statistically different from 100 mg/kg/day DCHP dose group (p=0.0024), ^E^ statistically different from 500 mg/kg/day DCHP dose group (p= 0.0009), ^F^ statistically different from control group (p=0.0095), ^G^ statistically different from control group (p <0.0001), ^H^ statistically different from control group (p=0.0021), ^I^ statistically different from 20 mg/kg/day DHP dose group (p <0.0001), ^J^ statistically different from 500 mg/kg/day DHP dose group (p=0.0460), ^K^ statistically different from 20 mg/kg/day DCHP dose group (p= 0.0096), ^L^ statistically different from 100 mg/kg/day DCHP dose group (p<0.0001), ^M^ statistically different from 500 mg/kg/day DCHP dose group (p<0.0001), ^N^ statistically different from 500 mg/kg/day DHP dose group (p=0.0015), ^O^ statistically different from 20 mg/kg/day DCHP dose group (p=0.2222).

Data were represented as mean ± SD of n=8-10 animals for each group. n: Number of male rats examined in each group.

The relative liver weight of female rats significantly increased in the 20 mg/kg/day DHP dose group compared to the control (p=0.0026) and 20 mg/kg/day DCHP dose groups (p=0.0345) (**Table 1**). In the male rats, relative liver weight significantly increased in the 100 mg/kg/day DCHP dose group compared to the control rats (p=0.0229) (**Table 1**). The relative kidney weight of male rats significantly decreased in the 20 mg/kg/day DHP (p<0.0001) and 500 mg/kg/day DCHP dose groups (p=0.0194) compared to the control rats (**Table 1**). On the other hand, in the female rats, relative kidney weight significantly increased in the 500 mg/kg/day DHP dose group compared to all other treatment and control groups except the 500 mg/kg/day DCHP-administered group (**Table 1**). Relative spleen weight of male rats significantly decreased in the 20 mg/kg/day DHP-treated group compared to all groups, including the control; however, in the female rats, relative spleen weight significantly decreased in the 20 mg/kg/day DHP dose group compared to the 100 mg/kg/day DHP-treated female rats (p=0.0044) (**Table 1**). In the female groups, relative stomach weight significantly decreased in the 100 mg/kg/day DCHP-administered rats compared to the 20 mg/kg/day (p=0.0457) and 100 mg/kg/day DHP dose groups (p= 0.0450), respectively (**Table 1**). On the other hand, relative stomach weight did not show any significant difference between the control and DHP or DCHP-treated groups (**Table 1**).

The relative heart weight of male rats significantly decreased in the 500 mg/kg/day DHP dose group compared to the 100 mg/kg/day DHP (p=0.0049) and DCHP dose groups (p=0.0189), respectively (**Table 1**). In the female rats, relative heart weight significantly increased in the 20 mg/kg/day DHP dose group compared to the 20 mg/kg/day DCHP-treated rats (p=0.0118); however considerably decreased in the 20 mg/kg/day DCHP dose group compared to the control (p=0.0130), 100 mg/kg/day DHP dose group (p=0.0470), 20 mg/kg/day DHP dose group (p<0.0001) and 100 mg/kg/day DCHP dose group (p=0.0010) (**Table 1**). Relative lung weight of male rats significantly decreased in the 20 mg/kg/day DHP-treated rats compared to the 500 mg/kg/day DHP (p=0.0280) and 100 mg/kg/day DCHP dose groups (p= 0.0083) (**Table 1**). In the female rats, relative lung weight significantly reduced in the 20 mg/kg/day DHP dose group compared to all DCHP dose groups (**Table 1**). Relative brain weight significantly decreased in 20 mg/kg/day DHP groups compared to the 100 mg/kg/day DCHP dose group (p=0.0372) in male rats and compared to the control group of female rats (p=0.0095) (**Table 1**). Relative brain significantly decreased in the 100 mg/kg/day DCHP dose group compared to the different from the control group (p <0.0001), 500 mg/kg/day DHP dose group (p=0.0015), and 20 mg/kg/day DCHP dose group (p=0.2222) in the female rats (**Table 1**).

### Evaluation of the serum biochemical parameters of male and female rats administered with DHP and DCHP

**Figure 1:**
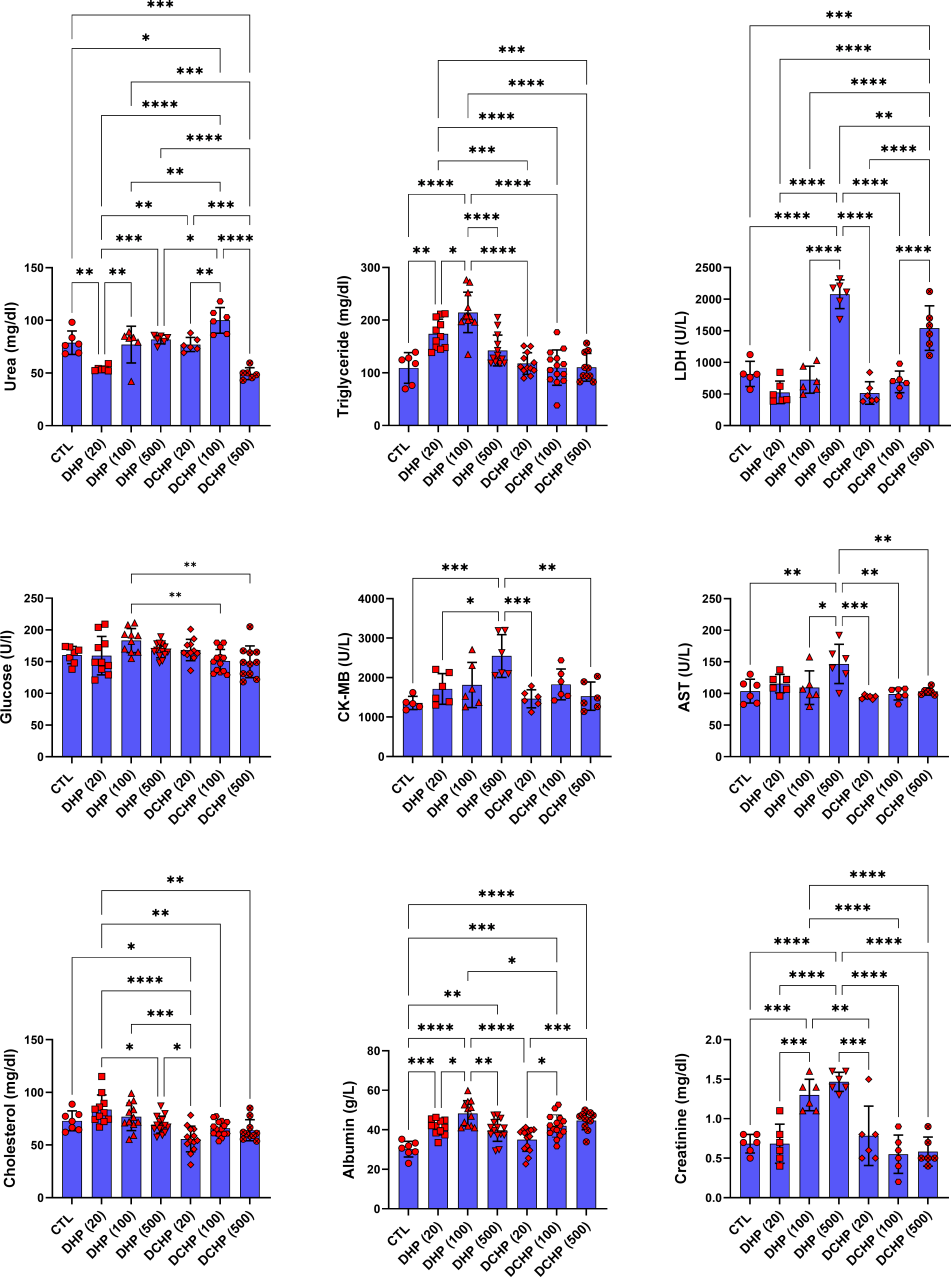
Serum biochemistry parameters were evaluated in adult male rats following in-utero DHP and DCHP administration at dosages 20, 100, and 500 mg/kg/day. Data were represented as the mean ± standard deviation (SD) following one-way analysis of variance (ANOVA) followed by Tukey’s *post hoc* test for multiple comparisons. Each group is compared to the control (CTL) group and each other, and a *p*-value ≤ 0.05 was considered significant for each data. *p ≤ 0.05, **p<0.01, ***p < 0.001, **** *p* < 0.0001, LDH, lactate dehydrogenase, CK-MB, creatine phosphokinase-MB, AST, aspartate aminotransferase.

**Figure 2:**
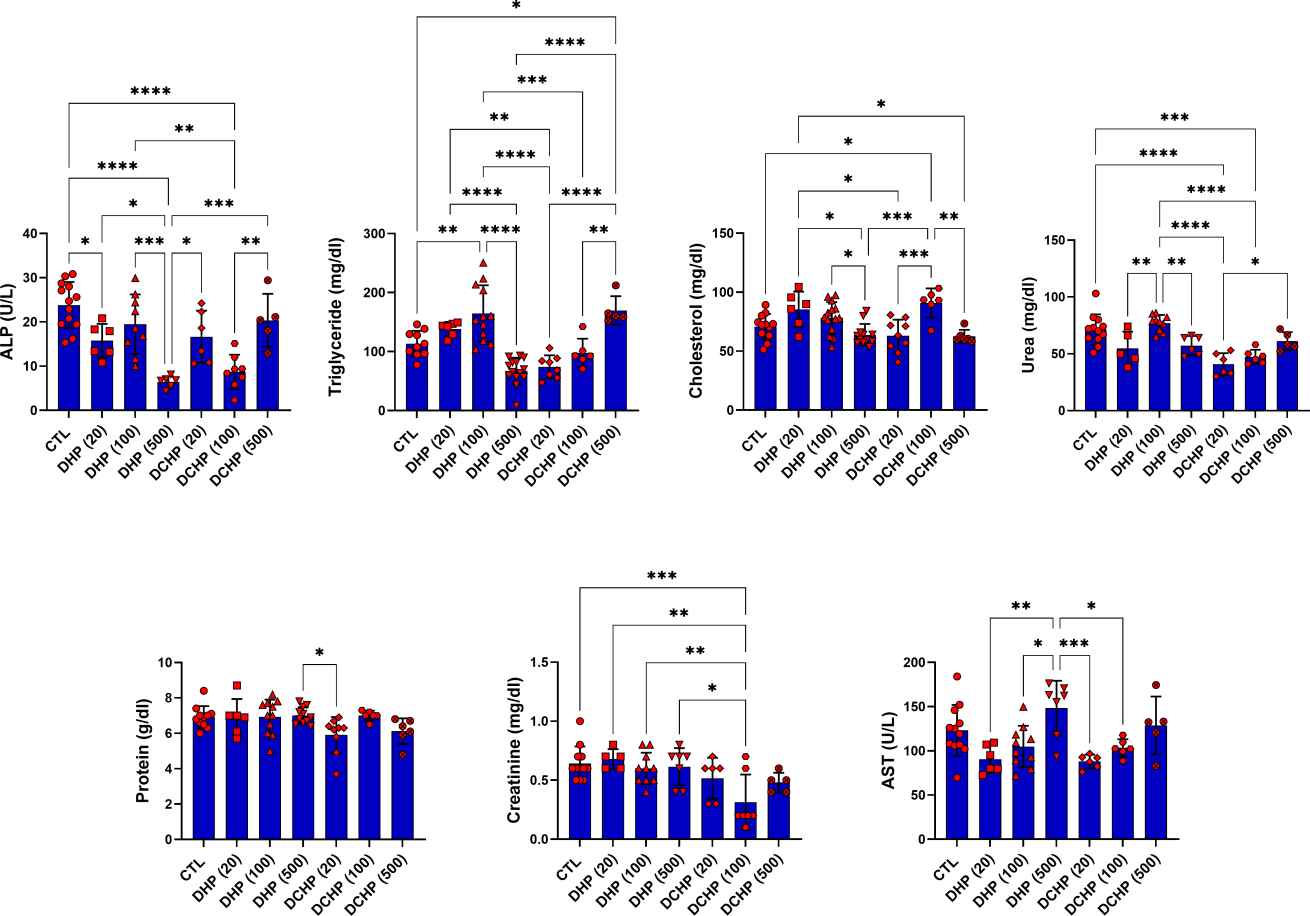
Serum biochemistry parameters were evaluated in adult female rats following in-utero DHP and DCHP administration at dosages 20, 100, and 500 mg/kg/day. Data were represented as the mean ± standard deviation (SD) following one-way analysis of variance (ANOVA) followed by Tukey’s *post hoc* test for multiple comparisons. Each group is compared to the control (CTL) group and each other, and a *p*-value ≤ 0.05 was considered significant for each data. *p ≤ 0.05, **p<0.01, ***p<0.001, ****p<0.0001, ALP, alkaline phosphatase, AST, aspartate aminotransferase.

Glucose, total protein, triglyceride, total cholesterol, albumin, AST, ALT, ALP, LDH, urea GGT, creatine kinase-myocardial band (CK-MB) levels were evaluated in the serum samples of DHP and DCHP-administered male and female rats. CK-MB and AST levels significantly elevated in 500 mg/kg/day DHP dose groups compared to the female control rats (**Figure 1**). Cholesterol levels significantly decreased in the 20 mg/kg/day DCHP dose group of female rats and 100 mg/kg/day DCHP dose group of male rats compared to the control rats, respectively (**Figures 1**, **2**). In the male rats, albumin concentration significantly elevated in all DHP and DCHP dose groups compared to the control rats except for 20 mg/kg/day DCHP-treated rats (**Figure 1**). Creatinine levels significantly increased in 100 and 500 mg/kg/day DHP dose groups of male rats; in contrast, they significantly decreased in 100 mg/kg/day DCHP-treated female rats compared to the control rats respectively (**Figures 1**, **2**). LDH concentration of male rats significantly elevated in 500 mg/kg/day DHP and DCHP dose groups compared to the control rats (**Figure 1**). Urea levels significantly decreased in 20 mg/kg/day DHP and 500 mg/kg/day DCHP dose groups, whereas they significantly increased in 100 mg/kg/day DCHP-treated male rats compared to the control group (**Figure 1**). In the male rats, triglyceride concentration elevated considerably in 20 and 100 mg/kg/day DHP dose groups compared to the control rats (**Figure 1**). Urea levels significantly decreased in 20 and 100 mg/kg/day DCHP-treated female rats compared to the control groups (**Figure 2**). Triglyceride levels significantly increased in 100 mg/kg/day DHP and 500 mg/kg/day DCHP dose groups compared to the control rats; on the other hand, ALP levels significantly decreased in 100 mg/kg/day DCHP, 20 and 500 mg/kg/day DHP dose groups compared to the control rats in female groups (**Figure 2**). LDH, glucose, CK-MB and albumin levels did not show any significant difference in the DHP and DCHP dose groups of female rats (**Figure 2**).

### Impact of the DHP and DCHP administration on the hematological parameters of male and female rats

**Figure 3:**
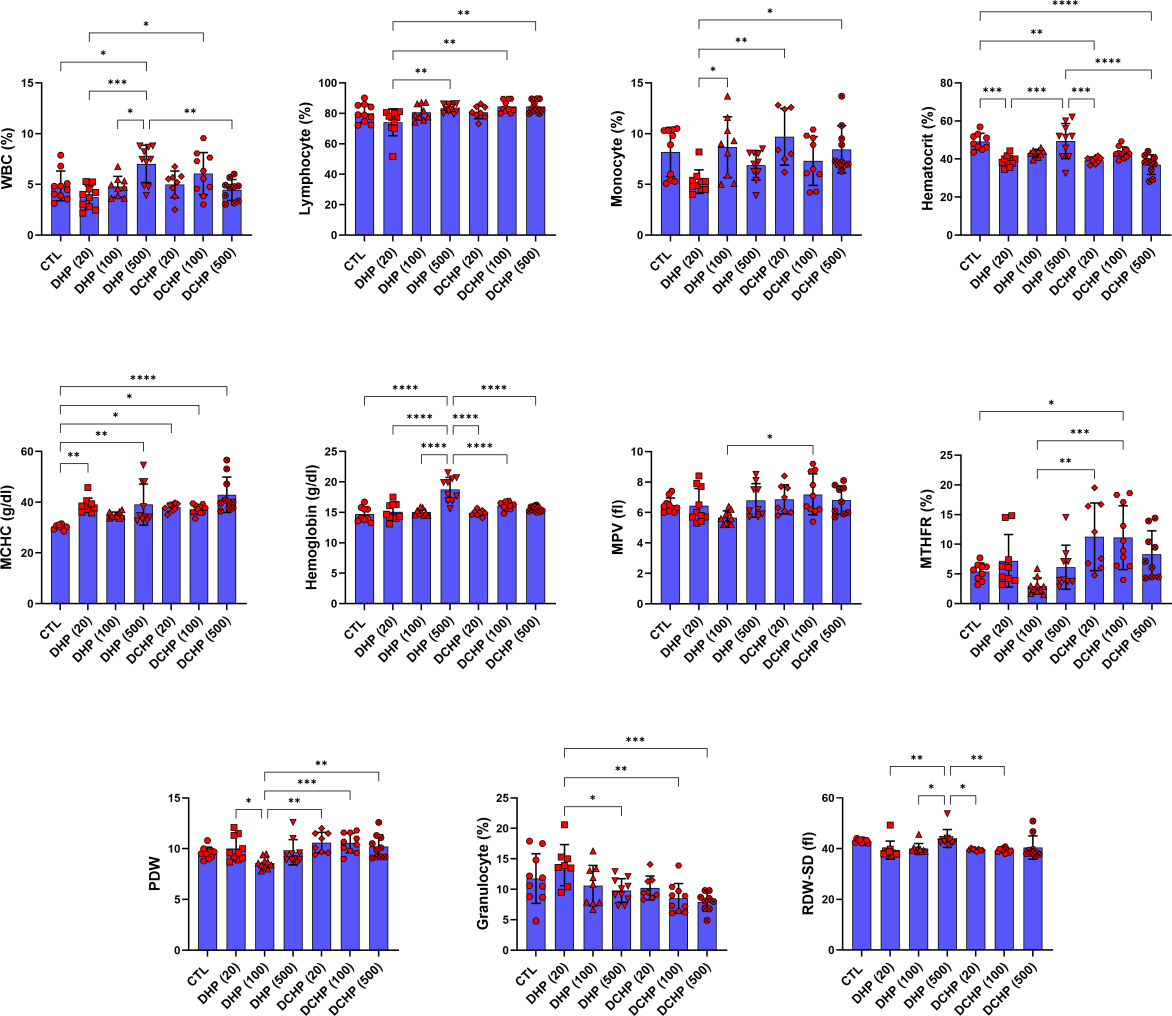
Hematological parameters were evaluated in adult male rats following in-utero DHP and DCHP administration at dosages 20, 100, and 500 mg/kg/day. Data were represented as the mean ± standard deviation (SD) following one-way analysis of variance (ANOVA) followed by Tukey’s *post hoc* test for multiple comparisons. Each group is compared to the control (CTL) group and each other, and a *p*-value ≤ 0.05 was considered significant for each data. *p ≤ 0.05, **p<0.01, ***p < 0.001, ****p < 0.0001, MCV, mean corpuscular volume, MCH, mean corpuscular hemoglobin, MCHC, mean corpuscular hemoglobin concentration; MPV, mean platelet volume; PDW, platelet distribution width, RDW, ratio distribution width of red blood cells, PCT, procalcitonin, RDW-SD, ratio distribution width of red blood cells-standard deviation, MTHFR, methylenetetrahydrofolate reductase, RDW-CV, red cell distribution width - coefficient of variation.

Leukocyte, lymphocyte, monocyte, granulocyte, erythrocyte, MCV, hematocrit, MCH, MCHC, hemoglobin, RDW-SD, RDW-CV, erythrocyte, platelet, MPV, PCT, PDW, and MTFHR were measured in male and female rats to evaluate effects of the DHP and DCHP administration on the hematological parameters. WBC and hemoglobin levels significantly increased in the 500 mg/kg/day DHP dose group compared to the control in male rats (**Figure 3**). MCHC levels significantly increased in all DHP and DCHP-treated groups of male rats compared to the control rats except 100 mg/kg/day DHP dose group (**Figure 3**). Hematocrit levels significantly decreased in the 20 mg/kg/day DHP, 20 mg/kg/day DCHP, and 500 mg/kg/day DCHP dose groups compared to the control rats in male groups. MTHR significantly increased in the 100 mg/kg/day DCHP-administered male rat compared to the control group (**Figure 3**).

**Figure 4:**
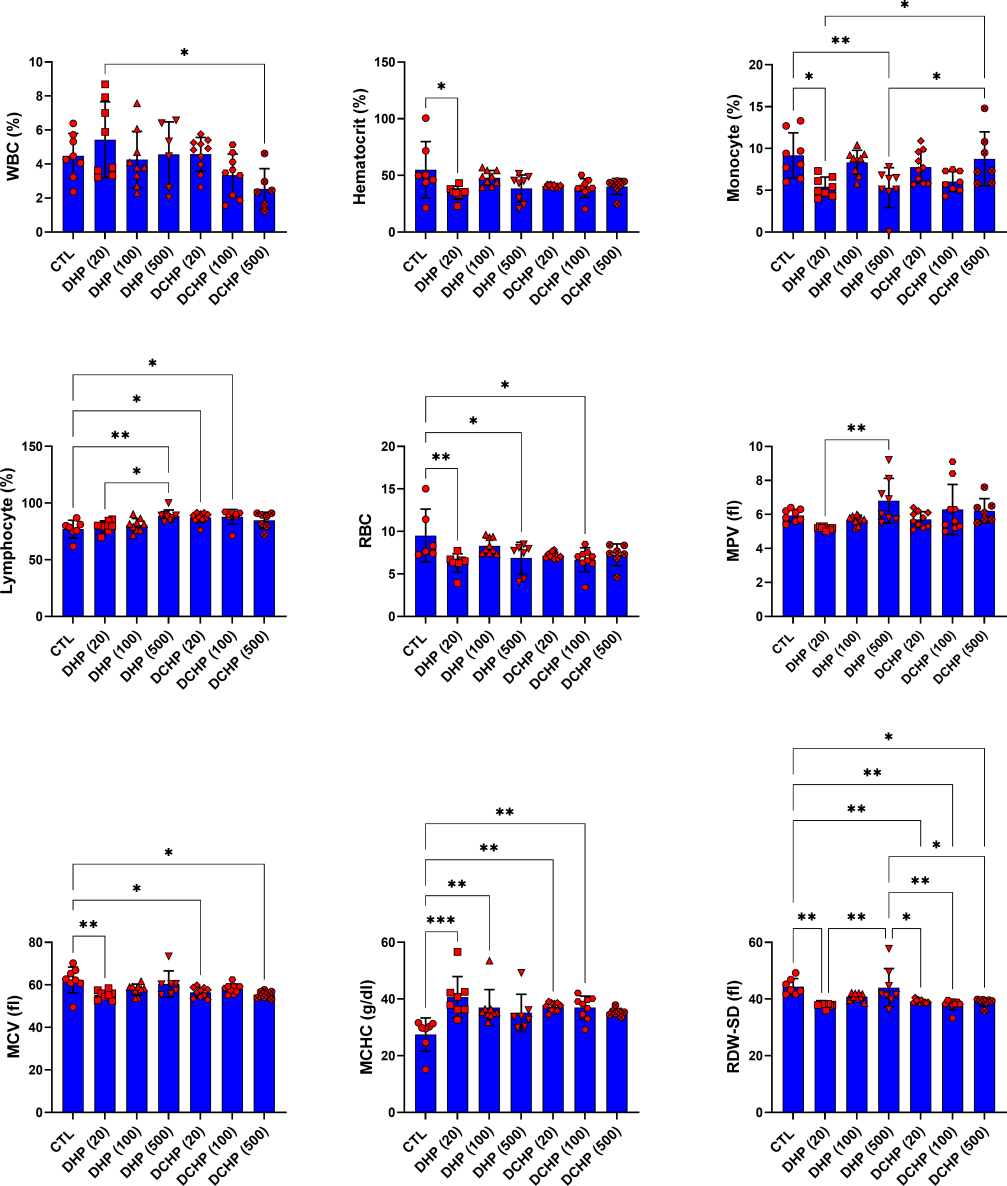
Hematological parameters were evaluated in adult female rats following in-utero DHP and DCHP administration at dosages 20, 100, and 500 mg/kg/day. Data were represented as the mean ± standard deviation (SD) following one-way analysis of variance (ANOVA) followed by Tukey’s *post hoc* test for multiple comparisons. Each group is compared to the control (CTL) group and each other, and a *p*-value ≤ 0.05 was considered significant for each data. *p ≤ 0.05, **p<0.01, ***p < 0.001, MCV, mean corpuscular volume, MCH, mean corpuscular hemoglobin, MCHC, mean corpuscular hemoglobin concentration; MPV, mean platelet volume; PDW, platelet distribution width, RDW, ratio distribution width of red blood cells, PCT, procalcitonin, RDW-SD, ratio distribution width of red blood cells-standard deviation, MTHFR, methylenetetrahydrofolate reductase, RDW-CV, red cell distribution width - coefficient of variation.

In the female rats, RBC and MCV levels significantly reduced in 20 mg/kg/day DHP, 500 mg/kg/day DHP, and 100 mg/kg/day DCHP dose groups compared to the female control rats (**Figure 4**). MCHC concentration in the female rats significantly elevated in the 10 mg/kg/day DHP and DCHP dose groups compared to the control rats (**Figure 4**). HCT concentration significantly decreased in the 20 mg/kg/day DHP dose group, and monocyte concentration significantly reduced in the 20 and 500 mg/kg/day DHP dose groups compared to the control, respectively (**Figure 4**). In the female rats, lymphocyte concentration significantly increased in the 500 mg/kg/day DHP, 20 mg/kg/day, and 100 mg/kg/day DCHP dose groups compared to the control rats (**Figure 4**). On the other hand, RDW-SD significantly reduced in 20 mg/kg/day DHP and all DCHP-treated female groups compared to the control rats (**Figure 4**). Hb, MTHFR, PDW and granulocyte levels did not significantly change in the female rats treated with DHP or DCHP, whereas RBC and MCV levels did not show any significant difference in DHP and DCHP administered male rats (**Figures 3**, **4**).

### DHP and DCHP-induced histopathological changes in the liver tissue of male and female rats

**Figure 5:**
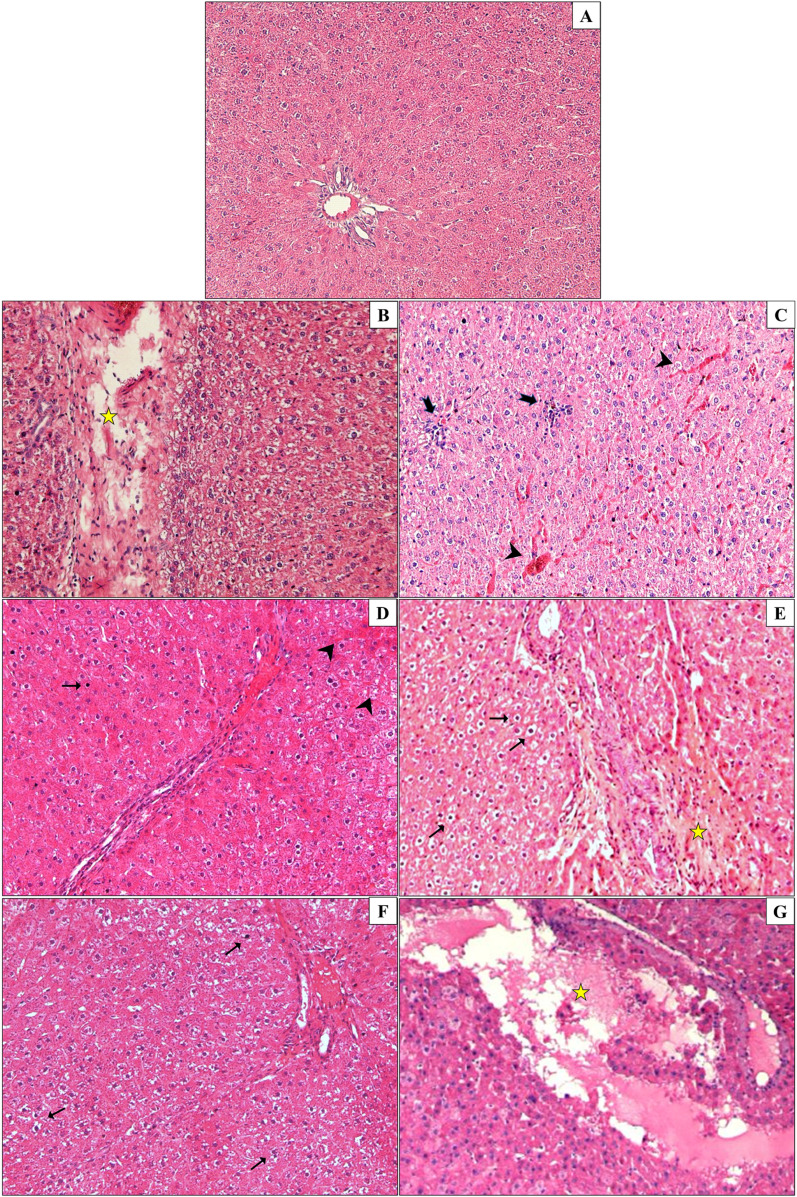
Liver samples of male and female rats were collected and stained with hematoxylin and eosin (H&E) staining to evaluate histopathological changes upon DHP and DCHP administration. Photomicrographs of adult rat liver sections in control **(A)**, 20 mg/kg/day DHP **(B)**, 100 mg/kg/day DHP **(C)**, 500 mg/kg/day DHP **(D)**, 20 mg/kg/day DCHP **(E)**, 100 mg/kg/day DCHP **(F)**, 5000 mg/kg/day DCHP **(G)** treated groups. In the liver tissues of DHP and DCHP-treated groups, congestion (arrowhead), inflammatory cell infiltration (thick arrow), cells with a pyknotic nucleus (thin arrow), lysis of hepatocytes, and degeneration of hepatic parenchyma (star) are shown. (magnification: x200).

Liver samples were collected and stained with H&E to evaluate histopathological changes in the liver. The incidence of exposure-associated histopathological lesions in the liver of adult female and male rats in the control and treatment groups is given in **Table 2**. Also, representative figures were chosen from H&E-stained liver slides and represented in **Figure 5**. Congestion, sinusoidal dilatation, inflammatory cell infiltration, cells with a pyknotic nucleus, lysis of hepatocytes, and degeneration of hepatic parenchyma have been observed in the liver samples of male and female rats exposed to the DHP and DCHP administration, respectively (**Table 2**, **Figure 5**). All the indicated histopathological changes significantly changed in all male and female rat liver samples of all DHP dose groups except 20 mg/kg/day DHP-treated groups compared to the control rats, respectively. Sinusoidal dilution and cells with pyknotic nucleus have not been considerably increased in the liver samples of the 20 mg/kg/day DHP and DCHP-treated male and female rats (**Table 2**). Inflammatory cell infiltration has risen significantly in the female liver samples of all DCHP dose groups compared to the control rats (**Table 2**). Considerable changes in the cells with pyknotic nuclei and lysis of hepatocytes have been found in 100 and 500 mg/kg/day DCHP dose groups compared to the female control rats (**Table 2**).

**Table 2:** Liver histopathologic lesions were represented for male and female rats following in-utero exposure to DHP or DCHP at dosages of 20, 100, and 500 mg/kg/day.

		DHP (mg/kg/day)			DCHP (mg/kg/day)		
	Control	20	100	500	20	100	500
MALE							
**(n)**	**10**	**10**	**10**	**10**	**8**	**10**	**10**
Congestion	0	6 ^a^	7 ^a^	9 ^a^	4 ^a^	2	5 ^a^
Sinusoidal dilatation	0	1	6 ^a^	7 ^a^	4 ^a^	2	4
Inflammatory cell infiltration	0	8 ^a^	7 ^a^	7 ^a^	6 ^a^	6 ^a^	7 ^a^
Cells with pyknotic nucleus	0	1	6 ^a^	8 ^a^	2	8 ^a^	7 ^a^
Lysis of hepatocytes	0	9 ^a^	7 ^a^	7 ^a^	4 ^a^	6 ^a^	6 ^a^
Degeneration of hepatic parenchyma	0	9 ^a^	7 ^a^	6 ^a^	4 ^a^	4	4
FEMALE							
**(n)**	**8**	**8**	**10**	**10**	**10**	**10**	**9**
Congestion	0	5 ^a^	7 ^a^	8 ^a^	4	2	4
Sinusoidal dilatation	0	1	5 ^a^	7 ^a^	4	2	4
Inflammatory cell infiltration	0	7 ^a^	7 ^a^	6 ^a^	6 ^a^	6 ^a^	7 ^a^
Cells with pyknotic nucleus	0	1	6 ^a^	7 ^a^	2	8 ^a^	6 ^a^
Lysis of hepatocytes	0	8 ^a^	7 ^a^	7 ^a^	4	6 ^a^	6 ^a^
Degeneration of hepatic parenchyma	0	8 ^a^	6 ^a^	6 ^a^	4	4	3

*n, number of male and female rats examined in each group. **^a^** Statistically different from control group, p<0.05 (Fisher’s exact test).

### Pentose phosphate pathway and oxidative stress metabolisms are impaired in liver samples of DHP and DCHP-treated male and female rats

**Figure 6:**
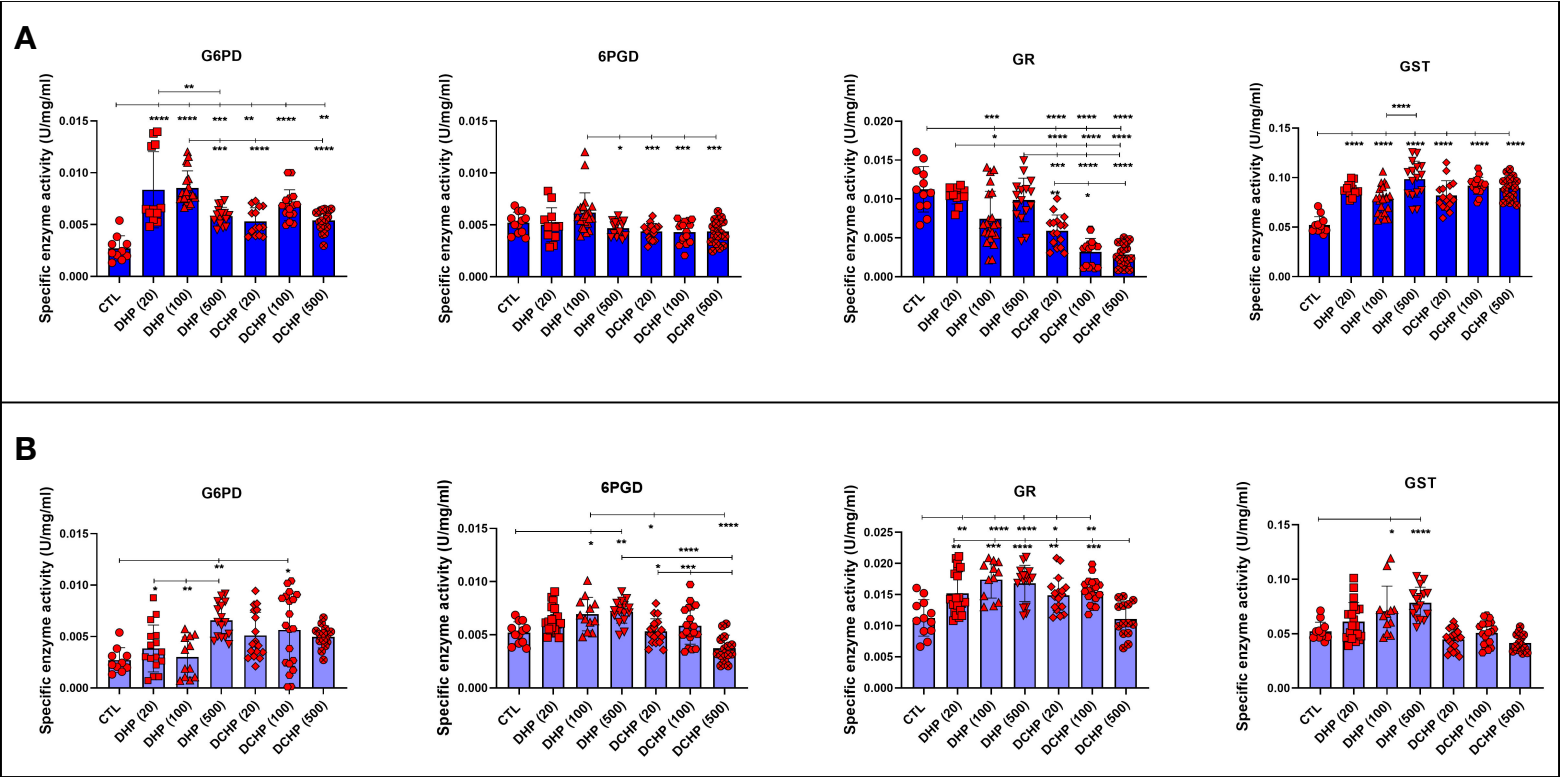
Oxidative stress and pentose phosphate pathway (PPP) metabolisms in the liver samples of male **(A)** and female rats **(B)** were evaluated *via* measuring glucose 6-phosphate dehydrogenase (G6PD), 6-phoshoglucanate dehydrogenase (6PGD), glutathione reductase (GR) and glutathione s-transferase (GST). Data were represented as the mean ± standard deviation (SD) following one-way analysis of variance (ANOVA) followed by Tukey’s *post hoc* test for multiple comparisons. Each group is compared to the control (CTL) group and each other, and a *p*-value ≤ 0.05 was considered significant for each data. *p ≤ 0.05, **p<0.01, ***p < 0.001, ****p < 0.0001.

**Figure 7:**
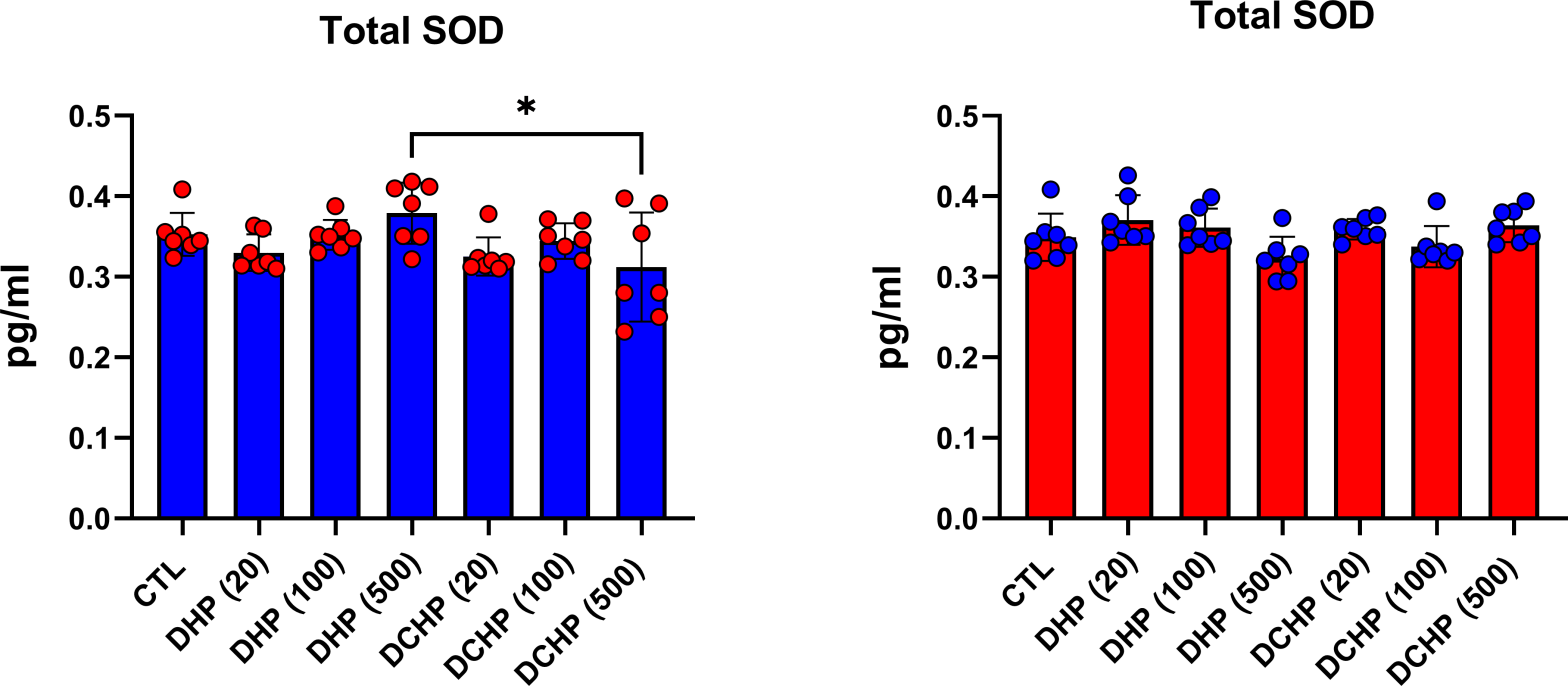
Superoxide dismutase (SOD) levels in the liver samples of male (blue) and female rats (red) were evaluated *via* ELISA assay. Data were represented as the mean ± standard deviation (SD) following one-way analysis of variance (ANOVA) followed by Tukey’s *post hoc* test for multiple comparisons. Each group is compared to the control (CTL) group and each other, and a *p*-value ≤ 0.05 was considered significant for each data. *p ≤ 0.05.

G6PD, 6PGD, GR, GST, and SOD enzymes were measured to evaluate oxidative stress metabolism and pentose phosphate pathway (PPP) in the liver samples following DHP and DCHP administration. G6PD levels increased in all DHP and DCHP-treated male rats compared to the control groups (**Figure 6A**). 6PGD activity was highest in the 100 mg/kg/day DHP-administered male rat liver compared to the control (**Figure 6A**). GR levels significantly decreased in 100 mg/kg/day DHP and all DCHP-administered groups compared to the control male rats; however, GST activities significantly elevated in all DHP and DCHP-treated male rats in comparison with control (**Figure 6A**). In the female rats, G6PD levels significantly elevated in the 500 mg/kg/day DHP and 100 mg/kg/day DCHP groups compared to the control, and the highest G6PD activity was observed in the 500 mg/kg/day DHP among all other treatment groups (**Figure 6B**). 6PGD and GST activities significantly increased in all DHP-treated female groups except the 20 mg/kg/day DHP group compared to control rats (**Figure 6B**). On the other hand, GR activity was significantly elevated in all DHP and DCHP-administered groups compared to the female control rats except in 500 mg/kg/day DCHP groups (**Figure 6B**). Total SOD levels were measured *via* ELISA, and SOD levels had no significant changes in DHP or DCHP dose groups compared to the control rats for both gender (**Figure 7**). However, in the male rats, SOD levels significantly decreased in the 500 mg/kg/day DCHP group compared to the 500 mg/kg/day DHP-treated group (**Figure 7**).

### DHP and DCHP administration increases FASN levels in rat liver

**Figure 8:**
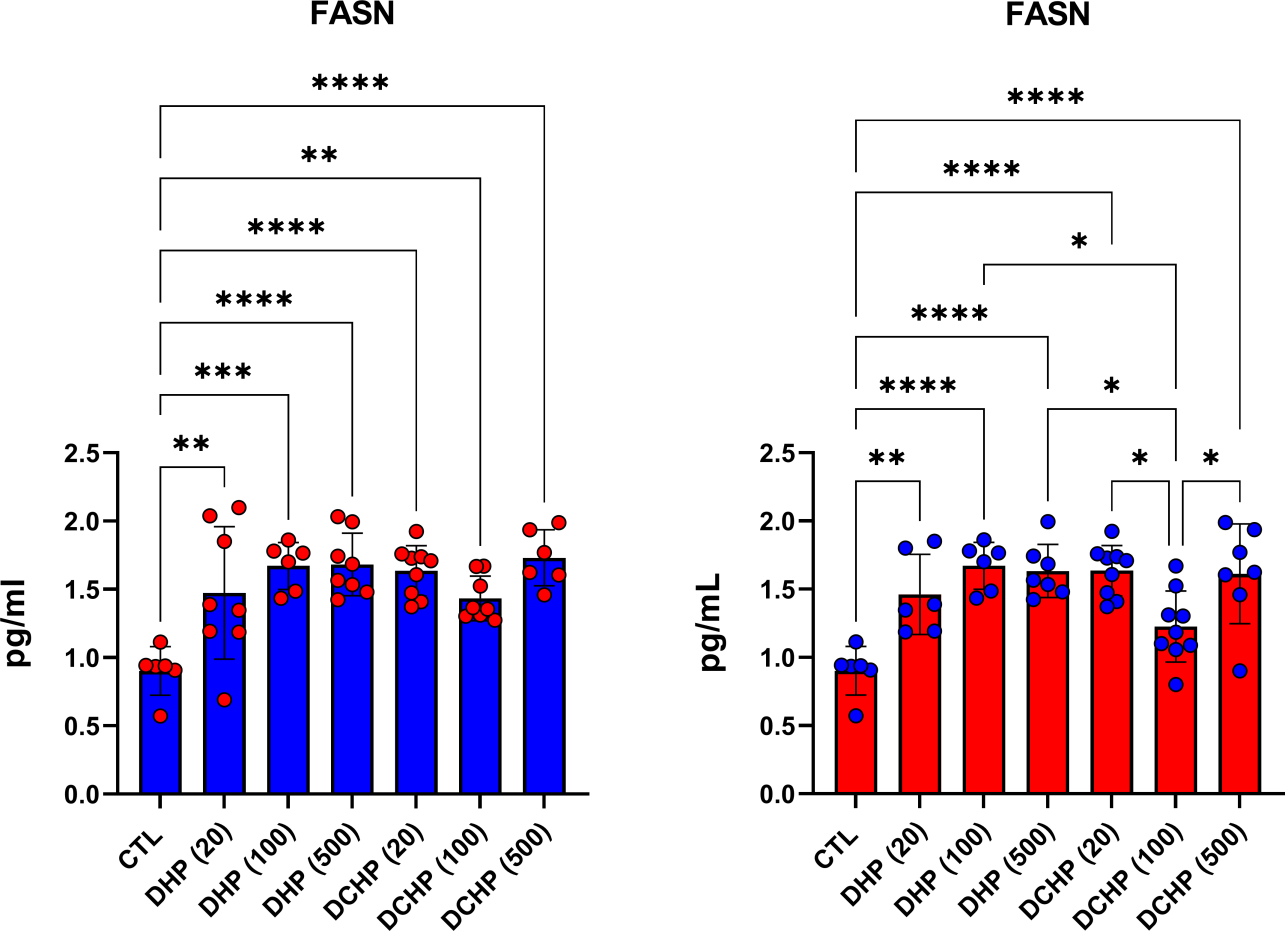
FASN levels in the liver samples of male (blue) and female rats (red) were evaluated *via* ELISA assay. Data were represented as the mean ± standard deviation (SD) following one-way analysis of variance (ANOVA) followed by Tukey’s *post hoc* test for multiple comparisons. Each group is compared to the control (CTL) group and each other, and a *p*-value ≤ 0.05 was considered significant for each data. *p ≤ 0.05, **p<0.01, ***p < 0.001, ****p < 0.0001.

FASN levels in rat livers were measured *via* ELISA to evaluate the impact of DHP and DCHP administration on lipid metabolism. FASN levels significantly increased in all DHP and DCHP dose groups compared to the control rats for both gender; however, this increase was not only significant in female rats treated with 100 mg/kg/day DCHP (**Figure 8**).

## Discussion

Phthalates are used in almost all industrial products as plasticizers, exerting endocrine-disrupting effects on humans and wildlife. There is an increasing concern against phthalate consumption since these chemicals can be quickly released from industrial products and found in water, soil, and air (21). Exposure to various types of phthalates contributes to the pathogenesis of multiple types of diseases, including diabetes, cardiovascular diseases, obesity, metabolic syndrome, endocrine disorders, infertility, and developmental toxicity; however, the impact of in-utero or oral exposure to DHP and DCHP on the liver metabolism has not been studied (22). DHP and DCHP are listed as substances of severe concern and reported as toxic to reproduction in the EU officially; also, both chemicals are considered the most potent phthalates (6).

People are exposed to phthalates, including DHP and DCHP, *via* dermal route, digestion, and inhalation; after exposure, 70% of them are metabolized in the liver, kidney, and gut. However, some of these chemicals remain in the body without metabolizing and accumulate over time; phthalates can diffuse into the lipid bilayer of cells and spread to neighboring tissues because of their lipophilic structure (23). In-utero, chronic, or acute exposure results in impaired oxidative stress metabolism in the liver associated with the impaired endocrine system, cardiovascular diseases, obesity, infertility, and diabetes, according to the literature (11–13). Thus, we investigated the impact of *in-utero* exposure to the DHP and DCHP on the oxidative stress-induced histopathological changes in the liver as the critical metabolic organ for energy and detoxification metabolisms first time in the literature (22).

Male and female rats were sacrificed at the postnatal day 90 following in-utero exposure to 20, 100, and 500 mg/kg/day DHP and DCHP (GD6 and GD19). The final body weight of the male rats did not show any significant difference between dose groups and control rats; however, body weight increased in the female rats in all DHP and DCHP dose groups compared to the female control rats except 100 mg/kg/day DHP dose group (**Supplementary Table 1**). Exposure to phthalates, especially at the prenatal stage, has been associated with an increased risk for obesity and body weight; thus, EDCs are referred to as obesogens, according to the literature (24). After sacrification, some organs were collected and weighed (**Supplementary Tables 1**, **2**) to calculate relative organ weight (mg/g). Evaluation of the relative organ weight changes is considered an effective indicator for chemically induced organ damage (25). In the female rats, relative liver weight increased in all DHP and DCHP dose groups compared to the control despite increased body weight (**Table 1**); however, a slight increase in the relative liver weight of male rats was observed in 20 and 100 mg/kg/day DCHP dose groups (**Table 1**). Increased relative liver weight has been associated with phthalate-induced tissue damage (26), and phthalate exposure resulted in increased liver weight in female rats rather than male rats, according to the literature (27).

ALT, AST, ALP, LDH, total protein, albumin, bilirubin, and globulin levels are biomarkers to evaluate liver function. ALT and AST participate in gluconeogenesis to produce oxaloacetate and pyruvic acid, respectively. Hepatocellular injury and cell death induced increased release into the circulation; on the other hand, elevated AST and ALT levels are associated with obesity and higher body mass (28, 29). ALP is a zinc metalloenzyme abundantly found in the bile, intestine, and placenta (30). Among indicated liver biomarkers, ALP, AST, albumin, and LDH are crucial indicators of liver function, and in case of hepatocyte alteration, these enzyme levels increase in the serum because of the altered permeability of the hepatocyte membrane (31). According to our data, AST levels reached maximum concentration in 500 mg/kg/day DHP dose groups compared to the control in male and female rats (**Figures 1**, **2**). In the female rats, ALP levels reduced upon DHP and DCHP administration, and the highest decrease was observed in the 500 mg/kg/day DHP dose group compared to the control (**Figure 2**). Increased AST levels are associated with liver damage and impaired mitochondria function; according to the literature, phthalate exposure increases AST levels (32, 33). ALP levels significantly decreased in DHP and DCHP dose groups of female rats, and this decrease was dramatic in the 500 mg/kg/day DHP dose group compared to the control rats (**Figure 2**). ALP is mainly found in the liver, and higher levels are associated with altered bone or liver metabolism; however, a dramatic decrease in ALP levels has been reported following estrogen and androgen therapy (34). First time in the literature, we have shown a dramatic reduction in the ALP levels upon phthalate exposure that may result from the estrogenic and androgenic properties of phthalates exerting endocrine-disrupting effects (35, 36).

Albumin is the most abundant plasma protein produced by the hepatic parenchymal cells, and lower albumin levels are correlated with liver disease (37). On the other hand, albumin regulates osmotic blood pressure and mediates the transportation of fatty acids, hormones, and drugs between tissues and organs. Increased binding ligands to the albumin are associated with altered ligand distribution, elimination, and metabolism (38). Phthalate exposure resulted in decreased albumin concentration, contrasting with our data that should be further investigated (39). Since LDH plays a vital role in energy metabolism, and higher serum levels result from tissue damage, its levels significantly increase in hepatocellular necrosis, lymphoma, and liver disease-induced hemolysis (38). Increased LDH activity has been reported upon phthalate activity, as shown in our data (**Figure 1**) (40). LDH levels significantly increased in the 500 mg/kg/day DHP and DCHP dose groups compared to the control in male rats (**Figure 1**). Albumin levels of male rats increased in all DHP and DCHP-administered groups compared to the control except the 20 mg/kg/day DCHP dose group (**Figure 1**).

In the male rats, glucose levels slightly increased in DHP dose groups compared to the control (**Figure 1**), and triglyceride concentration elevated in 20 and 100 mg/kg/day DHP dose groups compared to the control in male and female rats (**Figures 1**, **2**). According to the literature, the literature reported increased triglyceride levels and impaired lipid metabolism upon DEHP and DBP exposure (41, 42). The impact of phthalates on the glucose and lipid metabolism associated with obesity *via* the activation peroxisome proliferator-activated receptor (PPAR) family. Thus, phthalates are also called peroxisome proliferators (PPs) and relate to liver dysfunction (43). PPAR-α activates increased lipolysis in the liver and regulates glucose metabolism by increasing glucose levels in serum; on the other hand, PPAR-α activates fatty acid uptake and β-oxidation, resulting in enhanced respiratory activity in the mitochondria (44). Phthalates activate PPAR-α leading to the reregulating of liver energy metabolism associated with altered fatty acid metabolism (45). Increased triglyceride and glucose levels can be related to PPARs upregulation upon DHP and DCHP administration (43, 44). Serum creatinine and urea levels are considered renal and liver biomarkers. Urea is the product of metabolism, and decreased serum urea correlates with poor liver function, hepatic fibrosis, and malnutrition. Creatinine is used for the prognosis and priority in liver transplantation as a component of the Model for Endstage Liver Disease score (46). Increased creatinine levels have been reported upon DEHP administration in rats associated with renal dysfunction (47), and decreased urea levels have been reported in MEHP and MBP-administered rats in the previous studies (48). Urea levels significantly reduced in the 20 mg/kg/day DHP and 500 mg/kg/day DCHP dose groups compared to the control male rats (**Figure 1**). In the female rats, urea levels decreased in all DHP and DCHP dose groups compared to the female control rats except 100 mg/kg/day DHP group (**Figure 2**). Creatinine levels significantly increased in 100 and 500 mg/kg/day DHP dose groups compared to the female control rats (**Figure 1**).

According to the literature, exposure to phthalate, including DEHP and MEHP, causes altered hematological parameters correlated with anemia, inflammation, and coagulation dysfunction (49). Despite several studies, the impact of phthalate administration on hematological parameters remains unclear. RBC levels significantly decreased in 100 mg/kg/day DCHP, 20 and 500 mg/kg/day DHP dose groups compared to the control in female rats (**Figure 4**). Reduced RBC levels have been reported in phthalate-administered laboratory animals and increased urinary phthalate levels in humans correlated with decreased RBC count and anemia, associated with increased oxidative stress in the erythrocytes causing hemolysis (50). MCHC elevated in all DHP and DCHP dose groups compared to the control in male and female rats; however, hematocrit levels decreased in treatment groups compared to the control (**Figures 3**, **4**). Increased MCHC and decreased hematocrit levels have been reported in DEHP and dimethyl phthalate (DMP) administered rats associated with anemia (51).

Absolut organ weight, relative organ weight, and serum biochemistry parameters indicated liver dysfunction according to our data; therefore, we have performed the histopathological examination of H&E-stained liver samples. Each liver sample belonging to the male and female rats was examined following H&E staining, and liver lesions were scored under the microscope (**Table 2**). Representative images were taken and represented to report histopathological alterations in the liver upon DHP and DCHP administration, respectively (**Figure 5**). All histopathological lesions addressing liver damage, including congestion, sinusoidal dilatation, inflammatory cell infiltration, cells with a pyknotic nucleus, lysis of hepatocytes, and degeneration of hepatic parenchyma have been found in all DHP and DCHP dose groups of both genders (**Table 2**). The number of cells with pyknotic nuclei significantly increased in 100 and 500 mg/kg/day DHP and DCHP dose groups compared to the control rats of both gender (**Figure 5**, **Table 2**). The pyknotic nuclei in hepatocytes represent the nucleus fragmentation associated with cell death (52); also, the lysis of hepatocytes indicates cell death (53). Lysis of hepatocytes significantly increased in all DHP and DCHP dose groups of both gender (**Figure 5**, **Table 2**).

ALP, AST, albumin, and LDH are crucial indicators of liver function, and in case of hepatocyte alteration, these enzyme levels increase in the serum because of the altered permeability of the hepatocyte membrane (31). ALP, AST, and LDH mainly reside in the hepatocytes and are released upon liver damage to the blood. Increased ALP, AST and LDH levels have been observed in 500 mg/kg/day DCHP dose groups associated with lysis of hepatocytes and cells with pyknotic nucleus found in the histopathological evaluation (**Figures 1**, **2**, **Table 2**) (31). Congestion and sinusoidal dilatation in the liver is mainly associated with the altered hepatic venous flow associated with the edema in the liver, which leads to the increased ALP, AST and LDH levels reported in the literature (54). Congestion and sinusoidal dilatation has risen dramatically in all DHP-treated groups compared to the control of both gender (**Figure 5**, **Table 2**). The most significant histopathological changes in 100 and 500 mg/kg/day DHP dose groups in male and female rats correlated with increased LDH and AST levels in the same dose groups (**Figure 1**, **Figure 2**). Inflammatory cell infiltration, congestion, sinusoidal dilatation, and degeneration of hepatic parenchyma in the liver are mainly correlated with increased inflammation and oxidative stress (54). Indicated histopathological changes have been observed in the DHP and DCHP dose groups, which were significant in all DHP dose groups of both gender (**Figure 5**, **Table 2**). Therefore, we further investigated oxidative stress metabolism in the liver of DHP and DCHP-treated rats over antioxidant enzyme activities.

The liver mainly regulates xenobiotic, glucose, and lipid metabolism, and phthalate-induced liver damage has been correlated with impaired oxidative stress metabolism because of the activation of PPARs (31, 55). Oxidative stress is characterized by reduced antioxidant defense and increased reactive oxygen species (ROS) in the cell. Glucose 6-phosphate dehydrogenase (G6PD) and 6-phosphogluconate dehydrogenase (6-PGD) involve in the PPP, and the NADPH-production required for the activities of the glutathione-dependent enzymes, including glutathione reductase (GR) and glutathione s-transferase (GST). Since the GR enzyme converts GSSG to GSH, this enzyme regulates GSH/GSSH balance in the cell (56, 57). GSH is considered one of the most potent antioxidant agents and an effective scavenger of ROS. The role of EDCs-induced oxidative stress on tissue damage, including the liver, has been reported in previous studies (3, 58, 59). On the other hand, the SOD enzyme is responsible for the conversion of O^−2^ to water (H_2_O) and hydrogen peroxide (H_2_O_2_) (56).

G6PD levels increased in male and female liver samples in all DHP and DCHP dose groups except the 100 mg/kg/day DHP group of female rats (**Figures 6A, B**); however, 6-PGD levels significantly elevated in 100 and 500 mg/kg/day DHP dose groups compared to the female control rats (**Figure 6B**). G6PD is the rate-limiting step of the PPP, and increased G6PD and 6-PGD activities cause enhanced glycolysis and PPP (60). High G6PD activity in the liver has been reported upon DEHP administration in rats (61). Exposure to xenobiotics increases the synthesis of antioxidant molecules and enzymes, including GR, GST, GSH, SOD, G6PD, and 6-PGD. PPP is upregulated to respond to increased oxidative stress in the cell to produce more NADPH. Since enhanced, ROS contributes to the oxidative stress damage lipids, protein, and DNA, and upregulation of PPP enables cells to produce intermediates for nucleotide synthesis for DNA repair (62).

GR activity reduced in DHP and DCHP dose groups compared to the control in male and female rats. GST activity increased in all DHP and DCHP dose groups of male rats; however, this increase was observed in DHP-treated female rats (**Figures 6A, B**). GST is an antioxidant enzyme detoxing xenobiotics, including EDCs, phthalates, and parabens (57, 63). GST enzyme forms conjugate with xenobiotics by using GSH to eliminate them, and during this process, GSSG is formed. Since GR has a vital role in converting GSSG to GSH, reduced GR levels and increased GST activity cause enhanced oxidative stress in the male liver upon DHP and DCHP administration (64, 65). In the female rats, increased GR and GST levels can be correlated with enhanced oxidative stress metabolism in the liver (**Figure 6B**). Total SOD levels did not significantly change in the male and female rats upon DHP and DCHP administration (**Figure 7**). Our data have revealed that G6PD, 6PGD, GR, and GST enzyme activities significantly increased in the 100 and 500 mg/kg/day DHP and DCHP dose groups compared to the control rats in both genders to fight against oxidative stress (**Figure 6**). Histopathological changes indicating liver damage in the 100 and 500 mg/kg/day DHP and DCHP dose groups correlate with our oxidative stress data (**Figures 5**, **6**). Higher doses of DHP showed more significant histopathological and biochemical changes compared to the DCHP in both gender, according to our data (**Figures 1**–**7**).

We further evaluated FASN levels in the liver *via* the ELISA method. FASN catalyzes *de novo* synthesis of fatty acids for energy storage, membrane integrity, and secretion of triglycerides. FASN regulates fatty acid metabolism *via* the activation of PPAR-α and contributes to triglyceride anabolism and catabolism (66). FASN levels significantly increased in all DEHP and DCHP dose groups compared to the control rats of both gender and the highest FASN levels were found in 100 and 500 mg/kg/day DHP and DCHP dose groups (**Figure 8**). Previous studies reported that DEHP and MEHP increased FASN triglyceride levels in the liver *via* activation of PPAR-α; however, no data address *in-utero* exposure of DHP and DCHP on the liver FASN levels (67). Moreover, we found increased triglyceride levels in the 100 mg/kg/day DHP dose groups in male and female rats (**Figures 1**, **2**). Further studies can be conducted to reveal the impact of DHP and DCHP on lipid metabolism in the liver.

Indicated histopathological and biochemical changes in the liver can result from phthalate-induced PPAR-α activation since PPAR-α null mice did not develop liver toxicity but renal and testicular toxicity. In contrast, normal mice developed liver toxicity upon DEHP administration (68). PPAR-α is widely expressed in the liver and regulates lipid, glucose, and inflammation metabolisms as primary transcriptional regulators (68). Dysregulated activation of PPAR-alpha is associated with impaired mitochondrial homeostasis and structure, causing elevated levels of oxidative stress and enhanced respiration that may contribute to the disease pathogenesis, including cancer, metabolic syndrome, obesity, liver dysfunction, and cardiovascular diseases (69). PPAR-α activation in the prenatal and adulthood stages can be evaluated to reveal mechanisms behind oxidative stress-induced liver damage in further studies. First time in the literature, we showed the impact of *in-utero* exposure to DHP and DCHP administration on the liver damage associated with enhanced oxidative stress metabolism in male and female rats. Our data reveal the possible adverse effects of the DHP and DCHP on the liver; thus, phthalate production and consumption should be reduced and tightly regulated, especially for pregnant individuals.

## Conclusion

Phthalates exert adverse health effects *via* endocrine disrupting and peroxisome proliferator effects, leading to diabetes, metabolic syndrome, cardiovascular diseases, infertility, and cancer. DHP and DCHP have widely used phthalates worldwide; however, the impact of in-utero exposure to these chemicals on the liver function associated with oxidative stress has not been studied. First time in the literature, we showed that in-utero exposure to DHP and DCHP causes liver damage associated with impaired oxidative stress metabolism in male and female rats. Our data may guide researchers and governments to regulate and restrict phthalates in industrial products.

## Data availability statement

The original contributions presented in the study are included in the article/**Supplementary Material**. Further inquiries can be directed to the corresponding author.

## Ethics statement

The animal study was reviewed and approved by The Approval of Ethics Committee of Hacettepe University, with the number B.30.2.HAC.0.01.00.05 approved all experimental procedures and animal use.

## Author contributions

DA was responsible for writing the original manuscript, methodology, data analysis, data interpretation, and visualization. MA-A was responsible for the methods and writing the original manuscript. NB and NU were responsible for conceptualization, resources and supervision. All authors contributed to the article and approved the submitted version.

## Supplementary material

Supplementary Table 1 Body (g) and absolute organ weights (g) of adult female rats were calculated following sacrification to evaluate the impact of *in-utero* administration of DHP and DCHP at the dosages of 20, 100, or 500 mg/kg/day, respectively. Data were represented as mean ± SD of n=8-10 animals for each group. n: Number of male rats examined in each group. ^a^ Statistically different from control group (p=0.0375), ^b^ statistically different from control group (p= 0.0077), ^c^ statistically different from control group (p=0.0085), ^d^ statistically different from 20 mg/kg/day DHP dose group (p=0.0011), ^e^ statistically different from 100 mg/kg/day DCHP dose group (p<0.0001), ^f^ statistically different from 500 mg/kg/day DCHP dose group (p<0.0001), ^g^ statistically different from control group (p= 0.0002), ^h^ statistically different from control group (p= 0.0045), ^i^ statistically different from control group (p= 0.0030), ^j^ statistically different from 100 mg/kg/day DHP dose group (p=0.0013), ^k^ statistically different from 20 mg/kg/day DCHP dose group (p= 0.0258), ^l^ statistically different from 100 mg/kg/day DCHP dose group (p= 0.0247), ^m^ statistically different from 500 mg/kg/day DCHP dose group (p= 0.0165), ^n^ statistically different from control group (p= 0.0196), ° statistically different from control group (p=0.0079), ^p^ statistically different from control group (p<0.0001), ^q^ statistically different from 20 mg/kg/day DHP dose group (p=0.0003), ^r^ statistically different from 500 mg/kg/day DHP dose group (p<0.0001), ^s^ statistically different from 100 mg/kg/day DCHP dose group (p<0.0001), ^t^ statistically different from 500 mg/kg/day DCHP dose group (p<0.0001), ^u^ statistically different from 20 mg/kg/day DHP dose group (p<0.0017), ^v^ statistically different from 100 mg/kg/day DHP dose group (p=0.0028), ^w^ statistically different from 20 mg/kg/day DCHP dose group (p=0.0402), ^x^ statistically different from control group (p= 0.0104), ^y^ statistically different from 100 mg/kg/day DHP dose group (p=0.0364), ^z^ statistically different from 500 mg/kg/day DHP dose group (p=0.0012), ^A^ statistically different from control group (p<0.0001), ^B^ statistically different from control group (p=0.0003), ^C^ statistically different from control group (p<0.0001), ^D^ statistically different from control group (p=0.0001), ^E^ statistically different from control group (p=0.0040), ^F^ statistically different from 100 mg/kg/day DHP dose group (p=0.0233), ^G^ statistically different from control group (p= 0.0172), ^H^ statistically different from 100 mg/kg/day DCHP dose group (p=0.0069), ^I^ statistically different from 500 mg/kg/day DCHP dose group (p=0.0014), ^J^ statistically different from 100 mg/kg/day DCHP dose group (p=0.0023), ^K^ statistically different from 500 mg/kg/day DCHP dose group (p=0.0004), ^L^ statistically different from 100 mg/kg/day DCHP dose group (p=0.0091), ^M^ statistically different from 500 mg/kg/day DCHP dose group (p=0.0016), ^N^ statistically different from control group (p= 0.0018), ° statistically different from 100 mg/kg/day DHP dose group (p=0.0002), ^P^ statistically different from 500 mg/kg/day DHP dose group (p=0.0002), ^Q^ statistically different from 20 mg/kg/day DCHP dose group (p=0.0018).

Supplementary Table 2 Absolute organ weights (g) of adult male rats were calculated following sacrification to evaluate the impact of *in utero* administration of DHP and DCHP at the dosages of 20, 100, or 500 mg/kg/day, respectively. Data were represented as mean ± SD of n=8-10 animals for each group. n: Number of male rats examined in each group. ^a^ Statistically different from DHP 100 mg/kg/day dose group (p<0.05), ^b^ statistically different from the control group (p=0.012), ^c^ statistically different from the 500 mg/kg/day DHP dose group (p<0.05), ^d^ statistically different from 100 mg/kg/day dose DCHP group (p<0.0001), ^e^ statistically different from 500 mg/kg/day DCHP dose group (p=0.021), ^f^ statistically different from 500 mg/kg/day DCHP dose group (p<0.05), ^g^ statistically different from 100 mg/kg/day DCHP dose group (p=0.081), ^h^ statistically different from 100 mg/kg/day DCHP dose group (p<0.05), ^h^ statistically different from the control group (p=0.0024), ^j^ statistically different from 100 mg/kg/day DHP dose group (p=0.0044), ^k^ statistically different from 500 mg/kg/day DHP dose group (p=0.011), ^l^ statistically different from 500 mg/kg/day DCHP dose group (p=0.036).
