# Supplementary material for: Effects of the in-utero dicyclohexyl phthalate and di-n-hexyl phthalate administration on the oxidative stress-induced histopathological changes in the rat liver tissue correlated with serum biochemistry and hematological parameters
Source: Front Endocrinol (Lausanne). 2023 May 19;14:1128202. doi: 10.3389/fendo.2023.1128202 (PMC10235726; doi:10.3389/fendo.2023.1128202)
Supplement: Supplementary Table 1 — Body (g) and absolute organ weights (g) of adult female rats were calculated following sacrification to evaluate the impact of in-utero administration of DHP and DCHP at the dosages of 20, 100, or 500 mg/kg/day, respectively. Data were represented as mean ± SD of n=8-10 animals for each group. n: Number of male rats examined in each group. a Statistically different from control group (p=0.0375), b statistically different from control group (p= 0.0077), c statistically different from control group (p=0.0085), d statistically different from 20 mg/kg/day DHP dose group (p=0.0011), e statistically different from 100 mg/kg/day DCHP dose group (p<0.0001), f statistically different from 500 mg/kg/day DCHP dose group (p<0.0001), g statistically different from control group (p= 0.0002), h statistically different from control group (p= 0.0045), i statistically different from control group (p= 0.0030), j statistically different from 100 mg/kg/day DHP dose group (p=0.0013), k statistically different from 20 mg/kg/day DCHP dose group (p= 0.0258), l statistically different from 100 mg/kg/day DCHP dose group (p= 0.0247), m statistically different from 500 mg/kg/day DCHP dose group (p= 0.0165), n statistically different from control group (p= 0.0196), ° statistically different from control group (p=0.0079), p statistically different from control group (p<0.0001), q statistically different from 20 mg/kg/day DHP dose group (p=0.0003), r statistically different from 500 mg/kg/day DHP dose group (p<0.0001), s statistically different from 100 mg/kg/day DCHP dose group (p<0.0001), t statistically different from 500 mg/kg/day DCHP dose group (p<0.0001), u statistically different from 20 mg/kg/day DHP dose group (p<0.0017), v statistically different from 100 mg/kg/day DHP dose group (p=0.0028), w statistically different from 20 mg/kg/day DCHP dose group (p=0.0402), x statistically different from control group (p= 0.0104), y statistically different from 100 mg/kg/day DHP dose group (p=0.036 [file Table_1.docx]

|  |  |  |  |  | **DHP (mg/kg/day)** | | | | | | | | |  | **DCHP (mg/kg/day)** | | | | | | | | |
| --- | --- | --- | --- | --- | --- | --- | --- | --- | --- | --- | --- | --- | --- | --- | --- | --- | --- | --- | --- | --- | --- | --- | --- |
|  | **Control** | | |  | **20** | | | **100** | | | **500** | | |  | **20** | | | **100** | | | **500** | | |
|  |  |  |  |  |  |  |  |  |  |  |  |  |  |  |  |  |  |  |  |  |  |  |  |
|  |  |  |  |  |  |  |  |  |  |  |  |  |  |  |  |  |  |  |  |  |  |  |  |
| **n** |  | 8 |  |  |  | 8 |  |  | 10 |  |  | 10 |  |  |  | 10 |  |  | 10 |  |  | 9 |  |
| **Body weight (g)** | 172 | ± | 8 |  | 194 | ± | 10 ^a^ | 166 | ± | 16 ^d,e,f^ | 183 | ± | 13 |  | 185 | ± | 17 | 197 | ± | 15 ^b^ | 198 | ± | 13 ^c^ |
| **Absolute Weight (g)** |  |  |  |  |  |  |  |  |  |  |  |  |  |  |  |  |  |  |  |  |  |  |  |
| *Liver* | 5.85 | ± | 0.6 |  | 7.7 | ± | 0.7 ^g,j,k^ | 6.1 | ± | 1.5 ^l,m^ | 6.6 | ± | 0.7 |  | 6.4 | ± | 0.6 | 7.3 | ± | 0.8 ^h^ | 7.4 | ± | 0.8 ^i^ |
| *Kidney* | 0.61 | ± | 0.03 |  | 0.67 | ± | 0.04 | 0.58 | ± | 0.04 ^q^,^r,s,t^ | 0.68 | ± | 0.06 ^n^ |  | 0.63 | ± | 0.07 | 0.70 | ± | 0.04 ^o^ | 0.72 | ± | 0.09 ^p^ |
| *Spleen* | 0.41 | ± | 0.06 |  | 0.36 | ± | 0.08 | 0.37 | ± | 0.08 | 0.41 | ± | 0.06 |  | 0.40 | ± | 0.05 | 0.50 | ± | 0.06 ^u,v,w^ | 0.45 | ± | 0.05 |
| *Stomach* | 1.04 | ± | 0.06 |  | 1.25 | ± | 0.07 ^x,y,z^ | 1 | ± | 0.11 | 0.97 | ± | 0.13 |  | 1.09 | ± | 0.11 | 1.10 | ± | 0.22 | 1.18 | ± | 0.12 |
| *Heart* | 0.53 | ± | 0.03 |  | 0.63 | ± | 0.05 ^A^ | 0.51 | ± | 0.04 | 0.62 | ± | 0.03 ^B^ |  | 0.53 | ± | 0.03 | 0.65 | ± | 0.05 ^C^ | 0.63 | ± | 0.04 ^D^ |
| *Thymus* | 0.3 | ± | 0.04 |  | 0.4 | ± | 0.09 | 0.38 | ± | 0.05 | 0.42 | ± | 0.08 |  | 0.39 | ± | 0.06 | 0.41 | ± | 0.05 | 0.49 | ± | 0.1 ^E,F^ |
| *Lung* | 1.13 | ± | 0.02 |  | 1.02 | ± | 0.1 ^H,I^ | 1.03 | ± | 0.1 ^J,K^ | 1.06 | ± | 0.1 ^L,M^ |  | 1.1 | ± | 0.2 | 1.38 | ± | 0.1 | 1.43 | ± | 0.1^G^ |
| *Brain* | 1.74 | ± | 0.03 |  | 1.72 | ± | 0.05 | 1.76 | ± | 0.06 | 1.76 | ± | 0.07 |  | 1.74 | ± | 0.06 | 1.64 | ± | 0.01 ^N,O,P,Q^ | 1.71 | ± | 0.04 |

**Supplementary Table 1.** Body (g) and absolute organ weights (g) of adult female rats were calculated following sacrification to evaluate the impact of *in utero* administration of DHP and DCHP at the dosages of 20, 100 or 500 mg/kg/day respectively. Data were represented as mean ± SD of n=8-10 animals for each group. **n**: Number of male rats examined in each group

**Notes:** ^a^ Statistically different from control group (p=0.0375), ^b^ statistically different from control group (p= 0.0077), ^c^ statistically different from control group (p=0.0085), ^d^ statistically different from 20 mg/kg/day DHP dose group (p=0.0011), ^e^ statistically different from 100 mg/kg/day DCHP dose group (p<0.0001), ^f^ statistically different from 500 mg/kg/day DCHP dose group (p<0.0001), ^g^ statistically different from control group (p= 0.0002), ^h^ statistically different from control group (p= 0.0045), ^i^ statistically different from control group (p= 0.0030), ^j^ statistically different from 100 mg/kg/day DHP dose group (p=0.0013), ^k^ statistically different from 20 mg/kg/day DCHP dose group (p= 0.0258), ^l^ statistically different from 100 mg/kg/day DCHP dose group (p= 0.0247), ^m^ statistically different from 500 mg/kg/day DCHP dose group (p= 0.0165), ^n^ statistically different from control group (p= 0.0196), ^o^ statistically different from control group (p=0.0079), ^p^ statistically different from control group (p<0.0001), ^q^ statistically different from 20 mg/kg/day DHP dose group (p=0.0003), ^r^ statistically different from 500 mg/kg/day DHP dose group (p<0.0001), ^s^ statistically different from 100 mg/kg/day DCHP dose group (p<0.0001), ^t^ statistically different from 500 mg/kg/day DCHP dose group (p<0.0001), ^u^ statistically different from 20 mg/kg/day DHP dose group (p<0.0017), ^v^ statistically different from 100 mg/kg/day DHP dose group (p=0.0028), ^w^ statistically different from 20 mg/kg/day DCHP dose group (p=0.0402), ^x^ statistically different from control group (p= 0.0104), ^y^ statistically different from 100 mg/kg/day DHP dose group (p=0.0364), ^z^ statistically different from 500 mg/kg/day DHP dose group (p=0.0012), ^A^ statistically different from control group (p<0.0001), ^B^ statistically different from control group (p=0.0003), ^C^ statistically different from control group (p<0.0001), ^D^ statistically different from control group (p=0.0001), ^E^ statistically different from control group (p=0.0040), ^F^ statistically different from 100 mg/kg/day DHP dose group (p=0.0233), ^G^ statistically different from control group (p= 0.0172), ^H^ statistically different from 100 mg/kg/day DCHP dose group (p=0.0069), ^I^ statistically different from 500 mg/kg/day DCHP dose group (p=0.0014), ^J^ statistically different from 100 mg/kg/day DCHP dose group (p=0.0023), ^K^ statistically different from 500 mg/kg/day DCHP dose group (p=0.0004), ^L^ statistically different from 100 mg/kg/day DCHP dose group (p=0.0091), ^M^ statistically different from 500 mg/kg/day DCHP dose group (p=0.0016), ^N^ statistically different from control group (p= 0.0018), ^O^ statistically different from 100 mg/kg/day DHP dose group (p=0.0002), ^P^ statistically different from 500 mg/kg/day DHP dose group (p=0.0002), ^Q^ statistically different from 20 mg/kg/day DCHP dose group (p=0.0018)
