# Supplementary material for: Effects of the in-utero dicyclohexyl phthalate and di-n-hexyl phthalate administration on the oxidative stress-induced histopathological changes in the rat liver tissue correlated with serum biochemistry and hematological parameters
Source: Front Endocrinol (Lausanne). 2023 May 19;14:1128202. doi: 10.3389/fendo.2023.1128202 (PMC10235726; doi:10.3389/fendo.2023.1128202)
Supplement: Supplementary Table 2 — Absolute organ weights (g) of adult male rats were calculated following sacrification to evaluate the impact of in utero administration of DHP and DCHP at the dosages of 20, 100, or 500 mg/kg/day, respectively. Data were represented as mean ± SD of n=8-10 animals for each group. n: Number of male rats examined in each group. a Statistically different from DHP 100 mg/kg/day dose group (p<0.05), b statistically different from the control group (p=0.012), c statistically different from the 500 mg/kg/day DHP dose group (p<0.05), d statistically different from 100 mg/kg/day dose DCHP group (p<0.0001), e statistically different from 500 mg/kg/day DCHP dose group (p=0.021), f statistically different from 500 mg/kg/day DCHP dose group (p<0.05), g statistically different from 100 mg/kg/day DCHP dose group (p=0.081), h statistically different from 100 mg/kg/day DCHP dose group (p<0.05), h statistically different from the control group (p=0.0024), j statistically different from 100 mg/kg/day DHP dose group (p=0.0044), k statistically different from 500 mg/kg/day DHP dose group (p=0.011), l statistically different from 500 mg/kg/day DCHP dose group (p=0.036). [file Table_2.docx]

|  |  |  |  |  | **DHP (mg/kg/day)** | | | | | | | | |  | **DCHP (mg/kg/day)** | | | | | | | | |
| --- | --- | --- | --- | --- | --- | --- | --- | --- | --- | --- | --- | --- | --- | --- | --- | --- | --- | --- | --- | --- | --- | --- | --- |
|  | **Control** | | |  | **20** | | | **100** | | | **500** | | |  | **20** | | | **100** | | | **500** | | |
|  |  | | |  |  | | |  | | |  | | |  |  | | |  | | |  | | |
|  |  |  |  |  |  |  |  |  |  |  |  |  |  |  |  |  |  |  |  |  |  |  |  |
| **n** |  | 10 |  |  |  | 10 |  |  | 10 |  | 10 |  |  |  |  | 8 |  |  | 10 |  |  | 10 |  |
| **Body Weight (g)** | 256 | ± | 10 |  | 256 | ± | 7 | 261 | ± | 5 | 265 | ± | 7 |  | 247 | ± | 11 | 260 | ± | 6 | 267 | ± | 6 |
| **Absolute Weight (g)** |  |  |  |  |  |  |  |  |  |  |  |  |  |  |  |  |  |  |  |  |  |  |  |
| *Liver* | 9.5 | ± | 1.3 |  | 9.2 | ± | 1.2 | 9.6 | ± | 0.6 | 9.3 | ± | 0.9 |  | 9.3 | ± | 1.1 | 9.9 | ± | 1 | 9.7 | ± | 1 |
| *Kidney* | 0.94 | ± | 0.08 |  | 0.84 | ± | 0.08 **^a^** | 0.92 | ± | 0.07 | 0.94 | ± | 0.1 |  | 0.88 | ± | 0.1 | 0.93 | ± | 0.07 | 0.91 | ± | 0.1 |
| *Spleen* | 0.53 | ± | 0.06 |  | 0.37 | ± | 0.06 **^a,b,c,d,e^** | 0.47 | ± | 0.06 | 0.48 | ± | 0.08 |  | 0.48 | ± | 0.07 | 0.54 | ± | 0.07 | 0.50 | ± | 0.07 |
| *Stomach* | 1.34 | ± | 0.2 |  | 1.4 | ± | 0.09 | 1.35 | ± | 0.1 | 1.27 | ± | 0.1 |  | 1.22 | ± | 0.1 | 1.26 | ± | 0.1 | 1.36 | ± | 0.1 |
| *Heart* | 0.7 | ± | 0.08 |  | 0.76 | ± | 0.1 | 0.8 | ± | 0.05 | 0.70 | ± | 0.07 |  | 0.68 | ± | 0.08 | 0.78 | ± | 0.1 | 0.74 | ± | 0.05 |
| *Thymus* | 0.42 | ± | 0.07 |  | 0.41 | ± | 0.04 | 0.43 | ± | 0.1 | 0.48 | ± | 0.07 |  | 0.37 | ± | 0.07 | 0.48 | ± | 0.06 | 0.42 | ± | 0.07 |
| *Lung* | 1.40 | ± | 0.11 |  | 1.14 | ± | 0.04 **^f,g,h^** | 1.41 | ± | 0.05 | 1.63 | ± | 0.22 |  | 1.25 | ± | 0.05 | 1.61 | ± | 0.07 | 1.57 | ± | 0.07 |
| *Brain* | 1.86 | ± | 0.07 |  | 1.79 | ± | 0.1 | 1.84 | ± | 0.07 | 1.83 | ± | 0.07 |  | 1.80 | ± | 0.04 | 1.7 | ± | 0.04 **^i,j,k,l^** | 1.81 | ± | 0.08 |

**Supplementary Table 2.** Absolute organ weights (g) of adult male rats were calculated following sacrification to evaluate the impact of *in utero* administration of DHP and DCHP at the dosages of 20, 100 or 500 mg/kg/day respectively. Data were represented as mean ± SD of n=8-10 animals for each group. **n**: Number of male rats examined in each group

**Notes:** ^a^ Statistically different from DHP 100 mg/kg/day dose group (p<0.05), ^b^ statistically different from control group (p=0.012), ^c^ statistically different from 500 mg/kg/day DHP dose group (p<0.05), ^d^ statistically different from 100 mg/kg/day dose DCHP group (p<0.0001), ^e^ statistically different from 500 mg/kg/day DCHP dose group (p=0.021), ^f^ statistically different from 500 mg/kg/day DCHP dose group (p<0.05), ^g^ statistically different from 100 mg/kg/day DCHP dose group (p=0.081), ^h^ statistically different from 100 mg/kg/day DCHP dose group (p<0.05), ^h^ statistically different from control group (p=0.0024), ^j^ statistically different from 100 mg/kg/day DHP dose group (p=0.0044), ^k^ statistically different from 500 mg/kg/day DHP dose group (p=0.011), ^l^ statistically different from 500 mg/kg/day DCHP dose group (p=0.036)
